# Effectiveness of a Home-Based Counselling Strategy on Neonatal Care and Survival: A Cluster-Randomised Trial in Six Districts of Rural Southern Tanzania

**DOI:** 10.1371/journal.pmed.1001881

**Published:** 2015-09-29

**Authors:** Claudia Hanson, Fatuma Manzi, Elibariki Mkumbo, Kizito Shirima, Suzanne Penfold, Zelee Hill, Donat Shamba, Jennie Jaribu, Yuna Hamisi, Seyi Soremekun, Simon Cousens, Tanya Marchant, Hassan Mshinda, David Schellenberg, Marcel Tanner, Joanna Schellenberg

**Affiliations:** 1 Faculty of Infectious & Tropical Disease, London School of Hygiene & Tropical Medicine, London, United Kingdom; 2 Department of Public Health Sciences, Global health - Health systems and policy, Karolinska Institutet, Stockholm, Sweden; 3 Ifakara Health Institute, Dar es Salaam, Tanzania; 4 ILS Brothers, Dar es Salaam, Tanzania; 5 Institute of Global Health, University College London, London, United Kingdom; 6 Faculty of Epidemiology and Population Health, London School of Hygiene & Tropical Medicine, London, United Kingdom; 7 Tanzania Commission for Science and Technology (COSTECH), Dar es Salaam, Tanzania; 8 Swiss Tropical & Public Health Institute, Basel, Switzerland; 9 University of Basel, Basel, Switzerland; King's College London, UNITED KINGDOM

## Abstract

**Background:**

We report a cluster-randomised trial of a home-based counselling strategy, designed for large-scale implementation, in a population of 1.2 million people in rural southern Tanzania. We hypothesised that the strategy would improve neonatal survival by around 15%.

**Methods and Findings:**

In 2010 we trained 824 female volunteers to make three home visits to women and their families during pregnancy and two visits to them in the first few days of the infant’s life in 65 wards, selected randomly from all 132 wards in six districts in Mtwara and Lindi regions, constituting typical rural areas in Southern Tanzania. The remaining wards were comparison areas. Participants were not blinded to the intervention. The primary analysis was an intention-to-treat analysis comparing the neonatal mortality (day 0–27) per 1,000 live births in intervention and comparison wards based on a representative survey in 185,000 households in 2013 with a response rate of 90%. We included 24,381 and 23,307 live births between July 2010 and June 2013 and 7,823 and 7,555 live births in the last year in intervention and comparison wards, respectively. We also compared changes in neonatal mortality and newborn care practices in intervention and comparison wards using baseline census data from 2007 including 225,000 households and 22,243 births in five of the six intervention districts. Amongst the 7,823 women with a live birth in the year prior to survey in intervention wards, 59% and 41% received at least one volunteer visit during pregnancy and postpartum, respectively. Neonatal mortality reduced from 35.0 to 30.5 deaths per 1,000 live births between 2007 and 2013 in the five districts, respectively. There was no evidence of an impact of the intervention on neonatal survival (odds ratio [OR] 1.1, 95% confidence interval [CI] 0.9–1.2, *p* = 0.339). Newborn care practices reported by mothers were better in intervention than in comparison wards, including immediate breastfeeding (42% of 7,287 versus 35% of 7,008, OR 1.4, CI 1.3–1.6, *p* < 0.001), feeding only breast milk for the first 3 d (90% of 7,557 versus 79% of 7,307, OR 2.2, 95% CI 1.8–2.7, *p* < 0.001), and clean hands for home delivery (92% of 1,351 versus 88% of 1,799, OR 1.5, 95% CI 1.0–2.3, *p* = 0.033). Facility delivery improved dramatically in both groups from 41% of 22,243 in 2007 and was 82% of 7,820 versus 75% of 7,553 (OR 1.5, 95% CI 1.2–2.0, *p* = 0.002) in intervention and comparison wards in 2013. Methodological limitations include our inability to rule out some degree of leakage of the intervention into the comparison areas and response bias for newborn care behaviours.

**Conclusion:**

Neonatal mortality remained high despite better care practices and childbirth in facilities becoming common. Public health action to improve neonatal survival in this setting should include a focus on improving the quality of facility-based childbirth care.

**Trial Registration:**

ClinicalTrials.gov NCT01022788

## Editors' Summary

**Background:**

In 1990, 12 million children—most of them living in resource-limited countries—died before their fifth birthday. Faced with this largely avoidable loss of young lives, in 2000, world leaders set a target of reducing under-five mortality (deaths) to one-third of its 1990 level by 2015 as Millennium Development Goal 4 (MDG4); this goal, together with seven others, aims to eradicate extreme poverty globally. Progress towards reducing under-five mortality has been good. However, MDG4 has not been met because of slow progress in reducing neonatal mortality—death during the first 28 days of life. In sub-Saharan Africa, for example, mortality during the first five years of life fell by 47% between 1990 and 2012, whereas neonatal mortality fell by only 28%. Consequently, neonatal deaths now account for a greater proportion of global child deaths than in 1990, and every year there are still 3 million neonatal deaths.

**Why Was This Study Done?:**

Experts estimate that advising mothers during pregnancy and in the days following delivery (the early postpartum period) about good delivery and newborn care practices could prevent a quarter of neonatal deaths. WHO and the United Nations Children's Fund (UNICEF) therefore recommend that all mothers in areas with high neonatal mortality should receive two home visits during the early postpartum period to assess the newborn’s health and to counsel mothers on newborn care practices. In this cluster randomized trial, the researchers investigate the effect on neonatal care and survival in rural southern Tanzania, a low-resource setting, of a volunteer-led home-based counseling strategy that meets these recommendations. The neonatal mortality rate (NMR) has declined markedly in recent years in Tanzania but varies widely across the country. A cluster randomized trial randomly assigns groups of people (here, women living in different “wards” consisting of three or four villages) to receive alternative interventions and compares outcomes in the differently treated “clusters.”

**What Did the Researchers Do and Find?:**

The researchers estimated baseline neonatal mortality in five rural districts in southern Tanzania (35 deaths per 1,000 live births) from a 2007 census. In early 2010, they trained 824 female volunteers to deliver key counseling messages (including messages about handwashing with soap before delivery and early and exclusive breast feeding) and supporting messages such as the importance of childbirth in a health care facility. The volunteers were asked to make three home visits during pregnancy and two home visits shortly after delivery to women living in 65 intervention wards in six rural Tanzanian districts (a district not included in the baseline census was included in the trial); 67 comparison wards did not receive the intervention. A 2013 survey of households indicated that, in the intervention wards, 59% of women received at least one volunteer visit during pregnancy and 41% received at least one visit postpartum. Neonatal mortality reduced from 35 to 31 and 30 deaths per 1,000 live births in the intervention and comparison wards, respectively, in the five districts with baseline data. Newborn care practices reported by mothers were better in the intervention wards than in the comparison wards. For example, 42% and 35% of the women in the intervention and comparison wards, respectively, reported immediate breast feeding. Finally, childbirth in a health care facility increased from 41% in 2007 to 82% and 75% in 2013 in the intervention and comparison wards, respectively.

**What Do These Findings Mean?:**

These findings provide no evidence for an effect on neonatal survival of the home-based counseling strategy tested here even though the intervention improved newborn care practices. Notably, although many of the women in the intervention wards received antenatal visits from the trained volunteers, fewer women received early postpartum visits, a finding that could be partly responsible for the lack of effectiveness of the intervention. The accuracy of these findings may be limited by certain aspects of the trial design. For example, some of the women in the comparison areas may have inadvertently received part of the intervention (“leakage”). In addition, newborn care practises improved much in both, intervention and comparison areas, included a doubling of facility delivery. Nevertheless, the lack of an effect of the intervention on neonatal mortality despite a moderate effect on newborn care behaviours throws some doubt on the existing evidence base in support of home-based counselling as an intervention to reduce neonatal mortality. Finally, the finding that childbirth in a health care facility increased during the study period without a concomitant reduction in neonatal mortality highlights the need to improve the quality of facility-based childbirth care in rural southern Tanzania and, possibly, in other resource-limited settings.

**Additional Information:**

This list of resources contains links that can be accessed when viewing the PDF on a device or via the online version of the article at http://dx.doi.org/10.1371/journal.pmed.1001881.

- WHO provides information on global efforts to reduce child mortality and on ending preventable neonatal deaths (available in several languages); its 2009 recommendations, "Home Visits for the Newborn Child. A Strategy to Improve Survival," are available
- UNICEF works for children’s rights, survival, development, and protection around the world; it provides information about MDG4 and about child health in Tanzania; its website provides detailed statistics about child health; its Committing to Child Survival: A Promise Renewed—Progress Report 2014 addresses neonatal mortality
- The Millennium Development Goals 2015 Report is available
- The Healthy Newborn Network is an online community of more than 80 partner organizations that addresses critical knowledge gaps in newborn health
- More information about the trial described here is available

## Introduction

Every year, 3 million babies around the world die during their first 28 d of life. Despite major improvements in child survival in the past decade, neonatal mortality has declined slowly. In sub-Saharan Africa between 1990 and 2012, mortality during the first 5 y of life fell by 47%, from 177 to 98 deaths per 1,000 live births, but mortality in the first 28 d of life, which is the newborn or neonatal period, fell by only 28%, from 45 to 32 deaths per 1,000 live births. Around 44% of all child deaths now occur in the first 4 wk of life [1]. Millennium Development Goal 4—to reduce child mortality by two-thirds between 1990 and 2015—will not be reached without accelerated progress in reducing neonatal mortality.

The recent *Lancet* Every Newborn series supports community-based strategies to improve intervention coverage and reduce inequities [2]. In 2005, the *Lancet* Neonatal Survival series estimated that 12%–26% of neonatal deaths could be prevented by universal outreach and family-community care during the antepartum, peripartum, and postpartum period by promoting uptake of care and evidence-based newborn practices such as early and exclusive breastfeeding, thermal care, and clean cord care, among others [3]. In 2009, after trials in Asia showed dramatic effects of home-based counselling on neonatal survival [4–7], WHO and the United Nations Children's Fund (UNICEF) recommended two home visits in the early postpartum period in high-mortality settings to assess newborns and counsel mothers on newborn care practices [8]. The African evidence base for this strategy is limited to a single study [9].

In Tanzania, neonatal mortality has declined from around 29 to 21 deaths per 1,000 live births between 2005 and 2013 nationally [10], while wide variations in subnational estimates are described [11]. The health system has a pyramidal structure. Antenatal, intrapartum, and postpartum care is offered by a relatively dense network of primary and referral facilities [12]. The Tanzanian government is committed to the implementation and scale-up of a community health worker structure, making the effect of volunteer-based home counselling strategy on neonatal mortality of direct national relevance.

Here we report a cluster-randomised effectiveness trial of the effects of a volunteer-led, home-based counselling strategy—also called the Improving Newborn Survival in Southern Tanzania (INSIST) study—with three home visits in pregnancy and two in the first few days of life, on newborn care and neonatal survival in a population of over 1.2 million people.

## Methods

### Ethical Approval and Consent

The study was registered (www.clinicaltrials.gov NCT01022788) and approved by the review boards of Ifakara Health Institute, the Medical Research Coordinating Committee of the National Institute for Medical Research, Tanzania, Tanzania Commission for Science and Technology, and the London School of Hygiene and Tropical Medicine, United Kingdom. Written informed consent was sought from the household head. In 2013 we also sought written informed consent from interviewed women aged 18–49 y and assent from women aged 13–17 y.

### Trial Design and Participants

This cluster randomised trial used wards (groups of three-to-four villages), as randomisation units, in a study area comprising all 132 wards in the six districts of Mtwara Rural, Newala, and Tandahimba in Mtwara region and Lindi Rural, Ruangwa, and Nachingwea in Lindi region. The intervention aimed to reach all pregnant women in intervention wards. Most residents were subsistence farmers living in small settlements (subvillages). Cashew nuts were the main cash crop, with fishing common along the coast. Most houses had mud walls and thatched roofs. A network of 200 dispensaries and health centres as well as six hospitals provided care of varying quality [13–15]. Almost 90% of women live within 5 km of primary facilities [16].

### Home-Based Counselling Intervention

The home-based counselling strategy, branded *Mtunze Mtoto Mchanga*, which means “protect your newborn baby” in Swahili, was developed in 2008–2009. Formative work included a baseline household survey in 2007, qualitative enquiry including birth narratives and focus group discussions, and a rapid review of other volunteer programmes in the area. The strategy was designed in consultation with the Ministry of Health and members of the WHO, UNICEF, and professional organisations [17–20]. Key counselling messages were selected on the basis of the frequency of the behaviour in 2007 (Table 1), the feasibility of change, and the likely impact on survival on the basis of evidence published at the time [3,21]. They included hygiene during childbirth, early and exclusive breastfeeding, and extra care for low-birthweight babies, including skin-to-skin care (Table 2).

**Table 1:** Coverage of newborn care behaviours in the years prior to the 2007 and 2013 survey and changes between 2007 and 2013 restricted to the five districts with baseline data.

	Baseline Survey 2007 (results from 5 districts)					Impact Survey 2013 (results from 5/6 districts)							Percentage Point Change since Baseline (restricted to 5 districts)	
	Intervention		Comparison		Odds ratio (OR) (95% confidence interval [CI])	Intervention		Comparison		Difference	OR (95% CI) (results from 6 districts)	*p*-value	Intervention	Comparison
	*N*	%	*N*	%		*N* 5/6 districts	% 5/6 districts	*N* 5/6 districts	% 5/6 districts					
Prepared soap for delivery * (home deliveries)	6,641	84	6,508	84	1.0 (0.8–1.2)	1,238/1,435	90/89	1,697/1,907	82/81	8%	2.0 (1.5–2.5)	<0.001	↑6%	↓2%
Prepared money for delivery *	N/A	N/A	N/A	N/A	N/A	6,964/7,770	95/95	6,733/7,463	91/90	4%	1.9 (1.5–2.3)	<0.001	N/A	N/A
Had a plan in case of emergencies (home deliveries) ~	6,578	58	6,438	59	1.0 (09–1.1)	1,224/1,415	71/70	1,676/1,882	64/63	7%	1.4 (1.2–1.7)	<0.001	↑13%	↑5%
Antenatal care (at least four times) *	11,530	43	10,446	40	1.2 (1.0–1.4)	6,981/7,789	47/47	6,781/7,519	43/43	4%	1.2 (1.0–1.4)	0.044	↑4%	↑3%
Facility delivery *	11,666	43	10,577	38	1.1 (0.8–1.6)	7,005/7,820	82/82	6,813/7,553	75/75	7%	1.5 (1.2–2.0)	0.002	↑39%	↑37%
**Clean hands for home delivery** ~	**6,684**	**71**	**6,639**	**75**	**0.8 (0.6**–**1.1)**	**1,168/1,351**	**93/92**	**1,598/1,799**	**89/88**	**6%/4%**	**1.5 (1.0**–**2.3)**	**0.033**	**↑22%**	**↑14%**
Baby immediately covered (<5 min after delivery) #	11,662	27	10,569	27	1.0 (0.9–1.1)	5,054/5,627	47/48	4,846/5,405	45/46	2%	1.1 (1.0–1.2)	0.057	↑20%	↑18%
Baby not bathed before 6 h after birth #	11,662	29	10,569	30	1.0 (0.7–1.3)	6,377/7,083	92/91	6,177/6,799	82/80	10%/11%	2.7 (2.1–3.4)	<0.001	↑63%	↑52%
**Breastfed within 1 h of birth** #	**11,639**	**19**	**10,555**	**18**	**1.1 (0.9**–**1.2)**	**6,562/7,287**	**42/42**	**6,346/7,008**	**34/35**	**8%/7%**	**1.4 (1.3**–**1.6)**	**<0.001**	**↑23%**	**↑16%**
**Fed only breast milk in the first 3 d** **after delivery** #	**11,543**	**50**	**10,488**	**48**	**1.1 (0.8**–**1.4)**	**6,801/7,557**	**90/90**	**6,316/7,307**	**79/79**	**11%**	**2.2 (1.8**–**2.7)**	**<0.001**	**↑40%**	**↑31%**
Nothing put on the cord #	11,404	72	10,335	72	1.0 (1.9–1.2)	6,665/7,403	92/92	6,423/7,092	87/87	5%	1.8 (1.5–2.1)	<0.001	↑20%	↑15%
Babies born prematurely taken to hospital ^	N/A	N/A	N/A	N/A	N/A	213/251	34/31	203/236	31/30	3%/1%	1.0 (0.6–1.9)	0.920	N/A	N/A
Practiced any skin-to-skin care for prematurely born babies^	N/A	N/A	N/A	N/A	N/A	213/248	43/42	203/233	37/36	6%	1.3 (0.9–1.9)	0.200	N/A	N/A
Sick babies taken to health facility ¤	N/A	N/A	N/A	N/A	N/A	1,007/1,151	80/80	1,008/1,302	77/77	3%	1.1 (0.9–1.4)	0.255	N/A	N/A

Key behaviours marked in bold

* all women who reported a live birth

^~^ women who reported a live birth delivered at home

^#^ women who reported a live birth with the baby surviving at least 3 d

^^^ live-born baby who the mother reported was born prematurely

^¤^ mother reported any sickness of her live-birth baby in the first month of life. N/A, not available

**Table 2:** Behaviours promoted in home-based counselling, adapted from [26].

Visit	Timing	Key Behaviours	Additional Behaviours
1	As soon as pregnant woman identified	Information on importance of birth attendant washing hands and wearing gloves	Promotion of birth preparedness: facility delivery, saving money, clean cloths, soap, new blade for cutting and clean thread for tying cord, gloves for birth attendant
2	4 wk after visit 1	Promotion of early and exclusive breastfeeding	Promotion of birth preparedness (as in visit 1)
3	At the beginning of the 9th mo of gestation	Reinforcing early and exclusive breastfeeding practices, including breastfeeding position. In case of home birth, reinforcing the following: birth attendant should wash hands and wear gloves, identification of low-birth-weight babies using foot size as a proxy, immediate referral for very small or premature babies and those who do not cry, and skin-to-skin care for small babies.	Promotion of birth preparedness (as in visit 1); information on the importance of thermal care: immediate drying and wrapping and delayed bathing; information on danger signs in newborns. In case of home birth, the cord should be cut with a clean blade and tied with a clean thread.
4	Day of delivery	For home and facility births: observe and counsel on breastfeeding and remind women to practice exclusive breastfeeding. In case of home birth: identify low-birth-weight babies using foot size as a proxy, immediate referral for very small or premature babies, and skin-to-skin care for small babies	Check on thermal care and knowledge of danger signs and reinforce putting nothing on the cord
5	3rd d after delivery	Observe and counsel on breastfeeding and remind about exclusive breastfeeding	Reinforce putting nothing on the cord
Extra visits for small babies:			
First extra visit	Day after visit 5	Promotion of skin-to-skin care until the baby does not want to be carried skin to skin	
Second extra visit	Day after visit 6	Promotion of skin-to-skin care until the baby does not want to be carried skin to skin	

Because weighing scales were unlikely to be sustainable, we developed a screening tool using newborn foot size as a proxy for birth weight so that volunteers could identify low birthweight or premature babies born at home [22]. Home-based treatment of sepsis was not included in the advice of key national stakeholders, who felt it would be neither feasible nor necessary given the relatively dense network of primary facilities. Supporting messages included advice and information on childbirth in health facilities, birth preparedness, thermal care (immediate drying and wrapping and delayed bathing), cord cutting with a clean blade, tying with clean thread, dry cord care, and danger signs for sick newborns [23]. We hypothesised that improved hygiene during childbirth, early and exclusive breastfeeding, and better thermal care for low-birth-weight babies would lead to a measurable reduction in neonatal mortality [19]. Facility-based work to improve the quality of care in pregnancy and childbirth was implemented in only 24 of 200 facilities because of financial and human resource constraints, half in intervention and half in comparison areas [23]. Women in intervention and comparison groups received standard facility-based health care throughout the study.

During January–June 2010, 824 female volunteers were trained, two from each village. They were selected by their communities and trained for 6 d by council health management teams, who were trained in turn by regional health teams. Although some had experience of volunteering, those involved in other volunteer programme at the time were not eligible to apply. The volunteers were trained to visit women and their families three times in pregnancy and twice in the early postpartum period. We chose to emphasise counselling during pregnancy through three pregnancy visits rather than two because a 2009 field visit to a similar study in Ghana [24] suggested that high coverage of an early postnatal visit would be challenging.

The strategy was designed for large-scale implementation using existing community governance structures and the health system. Supervision and support was provided by local leaders known as village executive officers, as well as local health facility staff once a month [25]. Every 3–4 mo, volunteers met with supervisors and district health staff at a ward-level review meeting. In each ward, ten review meetings were held between June 2010 and July 2013. These meetings gave important opportunities to collect, summarise, and provide feedback on internal monitoring data on coverage of home visits for the volunteers, to build their skills and knowledge beyond the initial training, and to fine-tune the intervention. For example, given emerging findings on high facility delivery rates, we introduced birth notification slips for facility-based staff to give to mothers to inform the volunteers about the need for a home visit following birth in a health facility.

**Fig 1:**
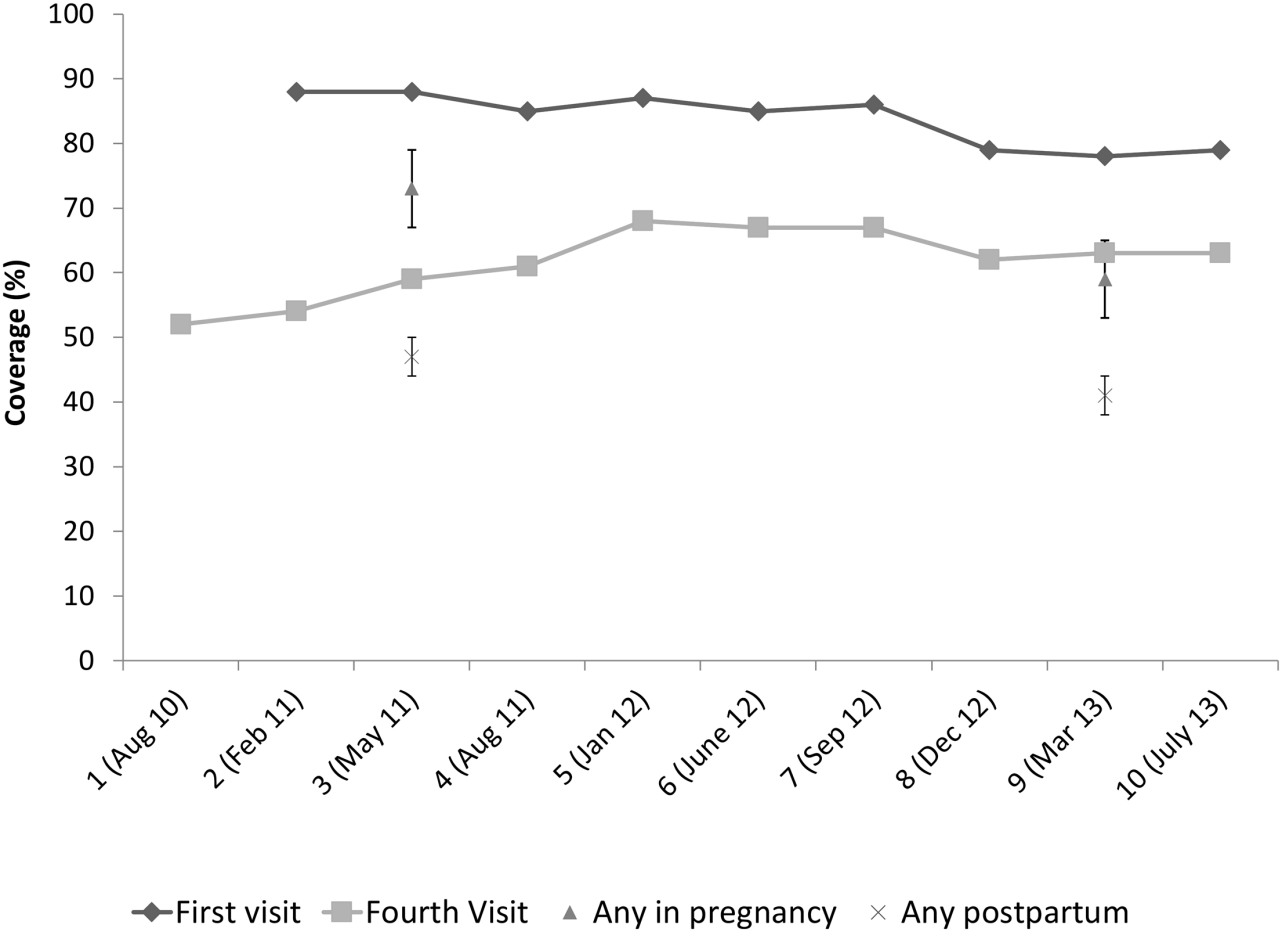
Estimated coverage of volunteer home visits from internal monitoring and household surveys. ~ Internal monitoring information (first and fourth visits) refers to information from the volunteers’ workbooks, which were collected throughout the study on a quarterly basis. The number of quarterly review meetings and the median month of data collection are given. * Household survey data include the adequacy survey done in 2011 (based on 257 women with a live birth in the year prior the survey [26]) and the impact evaluation household survey in 2013 (based on 7,823 women with a live birth in the year prior the survey).

We monitored volunteer visits recorded during quarterly review meetings and calculated coverage using expected births as a denominator; the expected births were calculated based on population data from 2007 and using a birth rate of 39 per 1,000 live births. These internal monitoring data suggested that of the 64,932 expected women with a live birth, approximately 80% in intervention areas had been visited by a volunteer in pregnancy and around 60% had been visited in the early newborn period (Fig 1, S1 Data). We did a follow-up survey including 5,000 households in 2011 to estimate implementation strength and changes in behaviour in intervention compared to comparison wards, to justify funding both for implementation until mid-2013 and for the 2013 end-line mortality survey. We saw improved newborn care behaviours such as the baby being bathed at least 6 h after birth (OR 2.0; 95% CI 1.2–3.4 comparing intervention and comparison areas) and exclusive breastfeeding for the first 3 d (OR 1.9; 95% CI 1.3–2.9) [26]. As a result, the international technical advisory board recommended that the impact evaluation was warranted.

### Outcomes

The primary outcome was the all-cause neonatal mortality rate (NMR) per 1,000 live births, defined as the proportion of all live births who died in the first 28 d of life (days 0–27), for children born between 1 July 2010 and 30 June 2013. Other mortality outcomes were days 1–27 mortality. The restriction to babies who survived the first 24 h after birth (day 0) was to exclude deaths due to intrapartum-related complications including asphyxia, for which the intervention was not expected to have a major impact.

The key behaviour (secondary) indicators were breastfeeding within an hour of delivery, prior handwashing with soap or use of gloves for those attending home deliveries, and exclusive breastfeeding for the first 3 d after birth. Other behaviour outcomes were skilled attendance at childbirth, birth preparedness, immediate drying and covering of the baby, clean cord care, delayed bathing, and identification and extra care for small babies, including skin-to-skin care for small babies and referral to hospital for very small babies. A data and safety monitoring board reviewed the study procedures and participant safety.

### Assessment of the Outcomes

Baseline neonatal mortality and newborn care practices were assessed through a survey of all households in five of the six study districts in 2007 (excluding Mtwara Rural) as part of a study assessing the effect of intermittent preventive treatment of malaria in infants [18,27]. In 2013 we did a representative household survey, including 185,000 households from all 132 wards. The primary sampling unit was the subvillage with a median of 100 households. As the study was randomised by ward and ward population varied from 1,000 to 22,000 people, we limited the sampling fraction in larger wards. We selected all subvillages in 56 smaller wards with 1,800 households or less and 20 subvillages from the 76 larger wards, chosen with probability proportional to size of the subvillage. Because of civil unrest, we reduced the sampling fraction to 11 subvillages in each ward of the Mtwara Rural district. Within the selected subvillages, we included all households if the local leaders estimated there were fewer than 130 households, which was the number that a single team of interviewers could manage in 1 d. For larger subvillages, we used segmentation to limit the sample to a maximum of 131 households. The survey sampled an estimated average of 94 households per subvillage.

With this sample size, we estimated that we would have complete data for at least 100 live births per ward per year in the 3 y leading to the survey, which would give 80% power to detect a 15% effect on neonatal mortality using a two-sided test at the 5% significance level assuming a coefficient of variation in ward mortality rates (SD/mean) of 0.21 and neonatal mortality of 34 per 1,000 in the comparison wards [28].

#### Data collection

In 2007 and 2013, data were collected by 22 and 20 teams, respectively, each with seven interviewers, a supervisor, a mapper/sensitiser, and a driver. An initial household listing module included the geographic location and the household head’s name. A household was defined as a group of people who live and eat together. If a household head refused to participate, replacement households were not approached. A household module included information on all members of the household, their dates of birth, education, and occupation; ethnic group of the household head; and asset ownership and housing characteristics as proxies of socioeconomic status. In a separate module, all resident, consenting women aged 18–49 y and assenting women aged 13–17 y were asked about live births in the 3 y prior to the survey, whether the child was still alive, and dates of all demographic events. For live births in the year before the survey, we asked about care in pregnancy, childbirth, and postpartum and about home counselling visits. We did not collect information on stillbirths.

#### Data collection and recording

In 2007 and 2013, all data were entered at the point of collection using personal digital assistants (PDAs, HP iPAQ HX2490 v6.1) programmed to allow internal range and consistency checks [29]. At the end of each module, each day, and once a week, data were backed up on electronic storage media. Quality control measures included accompanied interviews, random repeat interviews with follow-up action for discrepancies, supervisor visits to reportedly empty households, and daily reconciliation of handwritten summaries with computer-generated summaries before leaving the subvillage. The weekly reports summarised interviewer and team performance.

### Randomization and Masking

In 2009, 65 wards were randomised to the intervention, and 67 wards comprised comparison areas. Two wards with the same name were inadvertently randomised as one to the comparison group: they were analysed as two separate wards. To maximize balance between the two groups in the five districts with baseline data, we used implicit stratification with respect to district, division (an administrative structure between districts and wards), baseline NMR, and population. For the district without baseline data, we used implicit stratification by division [26]. Randomization was performed by JS using STATA. There were no exclusion criteria for clusters, households, or women. All wards agreed to participate, and volunteers were recruited from all intervention area villages. Consent to participate in the intervention was not formally sought from pregnant women, but they were free to refuse volunteer visits. Community members and health staff were not masked. The survey team was unaware of cluster allocation. The data analyst was masked to the cluster allocation until data cleaning was complete and a copy of the data lodged with the data and safety monitoring board.

### Analytical Methods

We used an intention-to-treat analysis, comparing children born to women in intervention and comparison wards according to a predefined analytical plan. We used random-intercept effects logistic regression using the *xtmelogit* command provided in Stata (Stata/IC Version 12.1), specifying the ward and subvillage level to account for the randomisation unit (ward level) and the clustered nature of the sample survey (subvillage level) [30].

The primary analysis was based on end-line data from all six districts. We computed odds ratios (OR) with 95% confidence intervals (CIs). We also estimated absolute risk differences for mortality.

We did secondary analyses of mortality for (1) the time periods of 2010–2011, 2011–2012, and 2012–2013 to investigate whether any effect changed over time; (2) babies surviving the first day of life (days 1–27); (3) facility and home births; (4) singletons; and (5) restricting the analysis to intervention wards with high home visit coverage, compared to all comparison wards. The analytical plan was reviewed and approved by the DSMB before data sets were “locked.” We repeated the analysis restricted to five districts and, as reviewers suggested, adjusted for baseline (2007) neonatal mortality, population size, and division. Based on reviewer advice, we also report a “per protocol” analysis in the web annex, although such results are likely to be biased (S2 Text). We estimated the effect of the intervention on newborn care behaviours using OR and percentage-point differences for newborn care practices.

We did not impute data for the district of Mtwara Rural, for which no baseline data were available. Multiple imputation would assume the information was missing at random. However, the Mtwara Rural district differs in numerous ways from the other five districts included in the study, for example, by not having a hospital. We also observed differences in poverty status and ethnic background.

We updated a meta-analysis conducted by Kirkwood and colleagues [9]. We searched PubMed using the terms (neonatal OR newborn) AND mortality AND trial and included those studies that examined the effect of community volunteers providing home visits during pregnancy or postpartum. No trials examining the effect of home visits on neonatal mortality other than those included in the 2013 systematic review of Kirkwood and colleagues [9] were retrieved. This included four proof-of-principle studies done in Southeast Asian countries [4–7] and a further four studies in a programme setting [9,31–33]. As most of the included studies presented risk ratios (RRs) and not ORs, we calculated the RR for NMR for our study using the margins from the random effects logistic model (marginal standardisation method) and estimated the CI via the delta technique [34]. As there was significant heterogeneity (>50%) between studies, we report the pooled result from a random effects meta-analysis.

## Results

**Fig 2:**
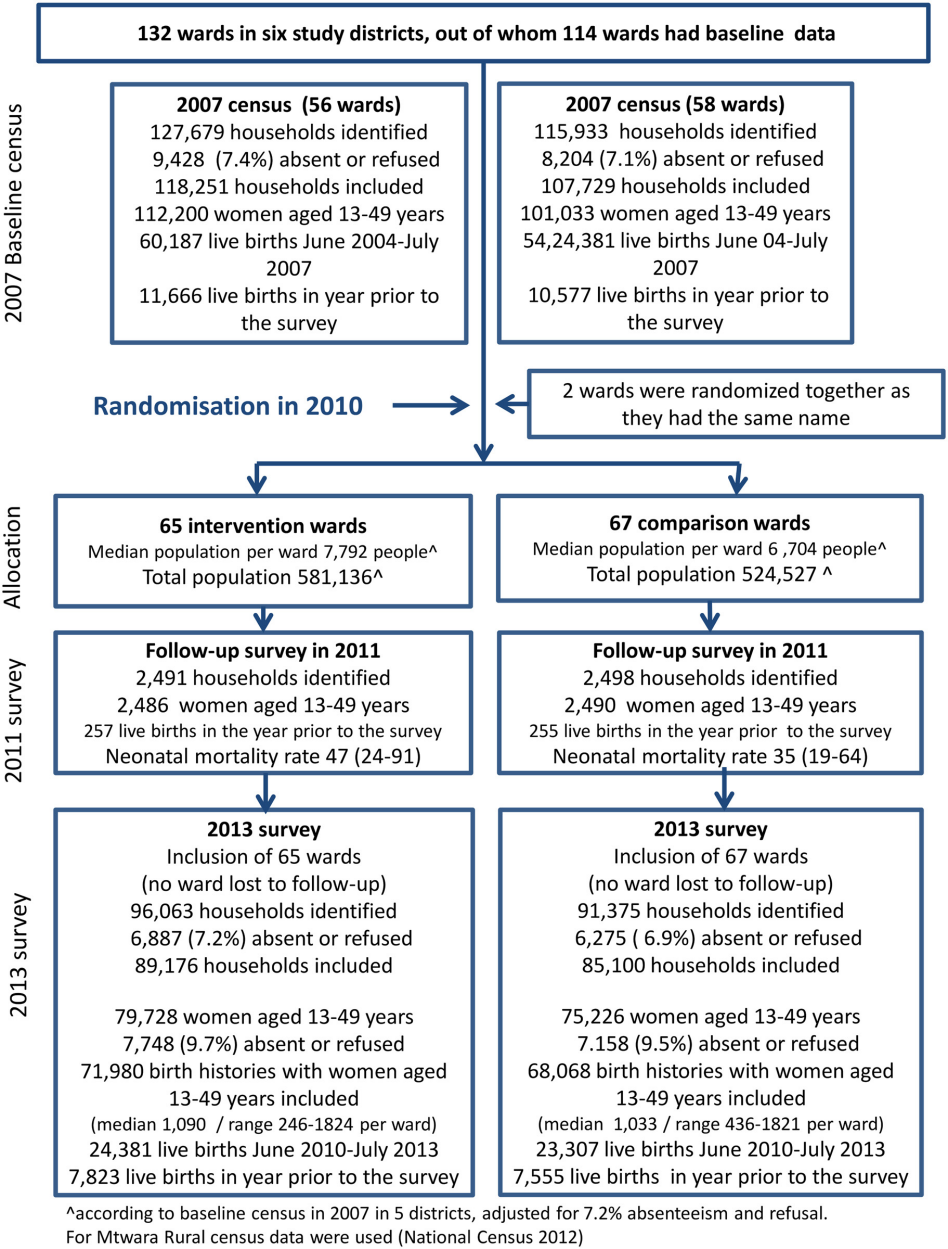
Trial profile.

The baseline survey in 2007 included all 243,612 households in five districts (except Mtwara Rural); 17,632 (7%) of the household heads were not present or refused participation. We interviewed 193,867 (91%) of 213,233 identified women of reproductive age (13–49 y); 22,243 had a live birth in the year prior to the survey (Fig 2, Table 3).

**Table 3:** Characteristics of respondents of intervention and comparison wards in the 2007 and 2013 surveys. The 2007 survey included the five districts of Lindi Rural, Ruangwa, Nachingwea, Newala, and Tandahimba. The 2013 survey additionally included the Mtwara Rural district.

	Intervention Wards 2007/2013 *n* = 127,679/96,063		Comparison Wards 2007/2013 *N* = 115,933/91,375		Percentage Point Difference 2007/2013
	*n*	%	*N*	%	%
**Household head present**	119,222/90,180	94/94	108,567/86,074	94/94	0/0
**Household head agreed to participate**	118,251/89,176	99/99	107,729/85,100	99/99	0/0
**Region**					
Lindi Region	65,989/45,655	56/51	60,776/43,165	56/51	0/0
Mtwara Region	52,262/43,521	44/48	46,953/41,935	44/49	0/-1
**Ethnic group**					
Makonde	63,259/49,546	54/56	60,836/49,730	56/58	-2/-2
Other	54,992/39,630	47/44	46,893/35,370	44/42	+3/+2
**Wealth quintiles (assets)**					
Most poor	21,900/15,999	19/18	20,846/16,565	19/20	0/-2
Very poor	19,855/17,374	17/20	18,385/16,856	17/20	0/0
Poor	24,597/16,891	21/19	18,178/15,956	21/19	0/0
Less poor	22,402/17,075	19/19	20,906/16,146	19/19	0/0
Least poor	23,008/18,723	20/21	20,078/16,567	19/20	1/1
Missing	6,489/3,114	6/4	5,336/3,010	5/4	1/0
**Maternal education**					
0–6 y of education	51,805/28,641	46/36	46,310/27,032	46/36	0/0
Completed primary education	59,609/50,523	53/63	53,993/47,698	53/63	0/0
Missing	786/564	1/1	730/496	1/1	0/0
**Maternal occupation**					
Farming	88,102/65,089	79/82	80,280/62,396	80/83	-1/-1
Other	4,721/3,785	4/5	3,751/2,924	4/4	0/1
Missing	19,377/10,854	17/14	17,002/9,906	17/13	0/0

No ward was lost to follow-up, and the data and safety monitoring board reported no safety concerns. In 2011, we did a follow-up survey in 5,000 households and estimated improved newborn care behaviours such as baby bathed at least 6 h after birth (OR 2.0; 95% CI 1.2–3.4 comparing intervention and comparison area) and exclusive breastfeeding for the first 3 d (OR 1.9; 95% CI 1.3–2.9) [26].

In 2013, six of 2,193 sampled subvillages refused to participate. A total of 187,438 households were visited between 1 July and 28 October 2013 (Fig 2, Table 3). In 11,184 (6%) households, no one was present; 1,978 (1%) refused to participate. We identified 154,954 women aged 13–49, of whom 140,048 (90%) agreed to be interviewed, 71,980 in the intervention wards and 68,068 in the comparison wards. These women reported 24,381 and 23,307 live births in the 3 y before the survey in intervention and comparison wards, respectively. Households and women were similar with regard to sociodemographic factors in the baseline and end-line surveys.

### Implementation strength

Reported coverage of at least one home-based counselling visit using the *Mtunze* job aids in pregnancy and postpartum was 59% (4,601 women) and 41% (3,216), respectively, in intervention areas compared with 4% (411 women) and 3% (259) in comparison areas (Table 4, Fig 1). Only 934 women (15%) in the intervention group and 51 (1%) in the comparison group reported a visit within 2 d postpartum after facility delivery (OR 21.7; 95% CI 14.6–32.2). Only 409 (5%) and 43 (1%) women reported receiving the full home visit schedule of three visits in pregnancy and two visits postpartum in intervention and comparison areas, respectively.

**Table 4:** Home counselling visit coverage reported by women with a live birth in the year prior to the 2013 survey.

	Intervention Wards		Comparison Wards		OR (95% CI)	*p*	Percentage Point Difference
	*n*/*N* = 7,823	%	*n*/*N* = 7,555	%			%
Home-based Counselling							
Women received a counselling visit during pregnancy*	4,601	59	411	4	41.5 (31.0–55.7)	<0.001	55
Women received a postpartum counselling visit*	3,216	41	259	3	28.6 (21.3–38.4)	<0.001	38
Women received a counselling visit* within 2 d of home delivery	275	19	7	0	75.0 (33.8–166.4)	<0.001	19
Women received a counselling visit within 2 d of facility delivery*	934	15	51	1	21.7 (14.6–32.2)	<0.001	14
Women received three visits in pregnancy and two postpartum, first within 2 d*	409	5	43	1	10.1 (7.1–15.8)	<0.001	4

* Volunteer who used the Mtunze counselling card or doll during the visit and where a card was left with the family

#### Primary mortality results

There was no evidence of an impact of the intervention on neonatal survival (31.6 versus 29.9 deaths per 1,000 live births in intervention and comparison wards, OR 1.1, 95% CI 0.9–1.2, *p* = 0.339) in the six districts (Table 5). Neonatal mortality reduced from 35.1 to 31.0 deaths per 1,000 live births in the intervention wards and from 34.9 to 30.0 in the comparison wards between 2007 and 2013 in the five districts, respectively. Analysis adjusted for baseline neonatal mortality, population, and division, restricted to the five districts when this was available, gave similar results (OR 1.0, 95% CI 0.9–1.2, *p* = 0.779). Neonatal mortality in the five districts where baseline data were available declined at 2% per year on average, from 35.0 (95% CI 33.5–36.5) to 30.8 per 1,000 live births (95% CI 29.3–32.5), a drop of 13% in 6 y.

**Table 5:** Primary analysis: Newborn mortality in intervention areas compared to comparison areas in 2007 and 2013 and adjusted for baseline.

Neonatal Mortality Rate per 1,000 Live Births	2007 (5 districts)				2013 (5/6 districts)			
	Intervention wards	Comparison wards	OR~ (95% CI)	*p*	Intervention wards	Comparison wards	OR~ (95% CI)	*p*
Live births	33,553	30,603			21,898 /24,381	21,085/23,307		
Deaths, days 0–27	1,140	1,035			661/749	616/679		
Neonatal mortality rate per 1,000 live births (95% CI)	35.1 (33.1–37.2)	34.9 (32.8–37.1)	1.0 (0.9–1.1)	0.830	31.0 (28.8–33.5)/ 31.6 (29.5–34.0)	30.0 (27.7–32.5)/ 29.9 (27.8–32.3)	1.0 (0.9–1.2)/1.1 (0.9–1.2)	0.547/0.339
Effect adjusted for baseline mortality and population size within the wards and division							1.0 (0.9–1.2)	0.779

^~^We used multilevel logistic regression to compute ORs specifying the ward and subvillage level. The intracluster correlation coefficients were 5.6% (95% CI 3.5%–8.8%) for the subvillage nested within the ward. The respective value for the ward level was 0.5% (95% CI 0.1%–2.7%).

#### Secondary mortality outcomes

There was no evidence of any difference in neonatal mortality between intervention and comparison groups of babies who survived the day of birth or for singleton babies (Table 6). The OR of dying in intervention compared with comparison wards was 1.1 (95% CI 0.9–1.3, *p* = 0.323) for babies who survived the day of birth and 1.1 (95% CI 0.9–1.2, *p* = 0.473) for the subgroup of singleton babies. Half of the neonatal deaths were on the first day of life (703 of 1,428, 49%), with no difference between intervention and comparison groups. We observed no difference in neonatal mortality between intervention and comparison areas when analysing the 3 y separately (2010–2011, 2011–2012, and 2012–2013) and thus no indication of a time trend.

**Table 6:** Secondary analysis, neonatal mortality in subgroups by intervention status, 2013 survey.

Neonatal Mortality Rate per 1,000 Live Births	Intervention Wards	Comparison Wards	Odds Ratio (95% CI)	*p*	Rate Difference
**All babies, post day 0 (day of birth)**					
Live births	23,966	22,922			
Deaths, days 1–27	334	294			
NMR per 1,000 live births (95% CI)	14.1 (12.7–15.7)	13.0 (11.6–14.5)	1.1 (0.9–1.3)	0.323	1
**Single births**					
Live births	23,760	22,714			
Deaths, days 0–27	665	611			
NMR per 1,000 live births (95% CI)	28.8 (26.6–31.0)	27.6 (25.5–29.9)	1.1 (0.9–1.2)	0.473	1
**Births in the year prior to the survey (July 2012–June 2013)**					
Live births	8,148	7,877			
Deaths, days 0–27	293	274			
NMR per 1,000 live births (95% CI)	37.3 (33.3–41.8)	36.0 (32.0–40.5)	1.0 (0.9–1.2)	0.674	1
**Births in the 2 y prior to the survey (July 2011–June 2012)**					
Live births	8,841	8,286			
Deaths, days 0–27	267	240			
NMR per 1,000 live births (95% CI)	31.0 (27.5–35.0)	29.8 (26.2–33.8)	1.04 (0.9–1.3)	0.645	1
**Births in the 3 y prior to the survey (Jul 2010–June 2011)**					
Live births	7,392	7,144			
Deaths, days 0–27	189	165			
NMR per 1,000 live births (95% CI)	26.2 (22.7–30.2)	23.6 (20.3–27.5)	1.11 (0.9–1.4)	0.348	2
**Analysis restricted to live births in the year prior to the survey with information on place of birth**					
**Live births in health facilities (any type)**					
Live births	5,309	4,686			
Deaths, days 0–27	186	153			
NMR per 1,000 live births (95% CI)	32.2 (27.7–37.2)	29.9 (25.5–35.0)	1.1 (0.9–1.4)	0.510	2
**Live births at home**					
Live births	1,240	1,654			
Deaths, days 0–27	38	57			
NMR per 1,000 live births (95% CI)	31.7 (23.1–43.6)	35.7 (27.5–46.2)	0.9 (0.6–1.3)	0.569	-4
**Restriction to intervention wards with coverage > 70% home visits** * **(all comparison wards included)**					
Live births	3,082	6,342			
Deaths, days 0–27	98	210			
NMR per 1,000 live births (95% CI)	32.8 (26.9–40.0)	34.2 (29.9–39.2)	1.0 (0.8–1.4)	0.749	-1
**Restriction to intervention wards with coverage > 80% home visits** * **(all comparison wards included)**					
Live births	1,095	6,342			
Deaths, days 0–27	36	210			
NMR per 1,000 live births (95% CI)	34.0 (24.5–47.1)	34.2 (29.9–39.2)	1.0 (0.7–1.4)	0.958	0

* Volunteer who used the Mtunze counselling card or doll during the visit

^#^ Volunteer visit in pregnancy and postpartum in which a Mtunze counselling card or doll was used during the visit

We found no evidence of any difference in neonatal mortality in babies born at home in intervention compared to comparison wards in the year prior to the survey (OR 0.9, 95% CI 0.6–1.3, *p* = 0.569) or in babies born in wards where the intervention had at least 70% coverage of at least one volunteer visit compared to comparison wards (OR 1.0, 95% CI 0.8–1.4, *p* = 0.749).

#### Newborn care behaviours (secondary outcomes)

There was strong evidence that the intervention improved coverage of key newborn care behaviours (Table 1) in the six districts. More women delivering at home had a birth attendant with clean hands (92% of 1,351 compared to 88% of 1,799; OR 1.5 95% CI 1.0–2.3, *p* = 0.033) comparing intervention and comparison wards of all six districts. More women reported breastfeeding within 1 h of birth (42% of 7,287 compared to 35% of 7,008; OR 1.4 95% CI 1.3–1.6, *p* < 0.001) and breastfeeding their babies exclusively for the first 3 d (90% of 7,557 compared to 79% of 7,307; OR 2.2, 95% CI 1.8–2.7, *p* < 0.001).

More women had emergency plans in case of home delivery (70% of 1,415 in intervention compared to 63% of 1,882 in the comparison areas; OR 1.4 95% CI 1.2–1.7, *p* < 0.001). More women reported delaying bathing their baby for six or more hours after birth (91% of 7,083 in intervention compared to 80% of 6,799 in comparison area; OR 2.7, 95% CI 2.1–3.4, *p* < 0.001). The proportion of sick babies taken to a health facility was similar in the two groups (80% of 1,151 and 77% of 1,302 in intervention and comparison groups, OR 1.1, 95% CI 0.9–1.4, *p* = 0.255). Coverage rates were virtually identical when restricting the analysis to five districts.

Comparison of key newborn care behaviours between 2007 and 2013 in intervention and comparison areas, for the five districts with baseline data, indicated relative large increases between 2007 and 2013 for clean hands for home delivery (22% compared to 14% increase in intervention and comparison wards, respectively), breastfeeding within 1 h (23% compared to 16% increase), and exclusive breastfeeding (40% compared to 31% increase).

Delivery in a health facility was 41% of 22,243 in 2007 and increased to 82% of 7,820 in the intervention area and 75% of 7,553 in the comparison area, a 39% and 37% point increase between 2007 and 2013 in intervention and comparison areas, respectively. Half of the facility births in 2013 took place in a hospital (5,937; 39%), and the other half in primary facilities: health centres (1,379; 9%) and dispensaries (4,715; 31%). In 2007, only 11% of 22,243 mothers delivered in primary facilities, and 29% in a hospital.

**Fig 3:**
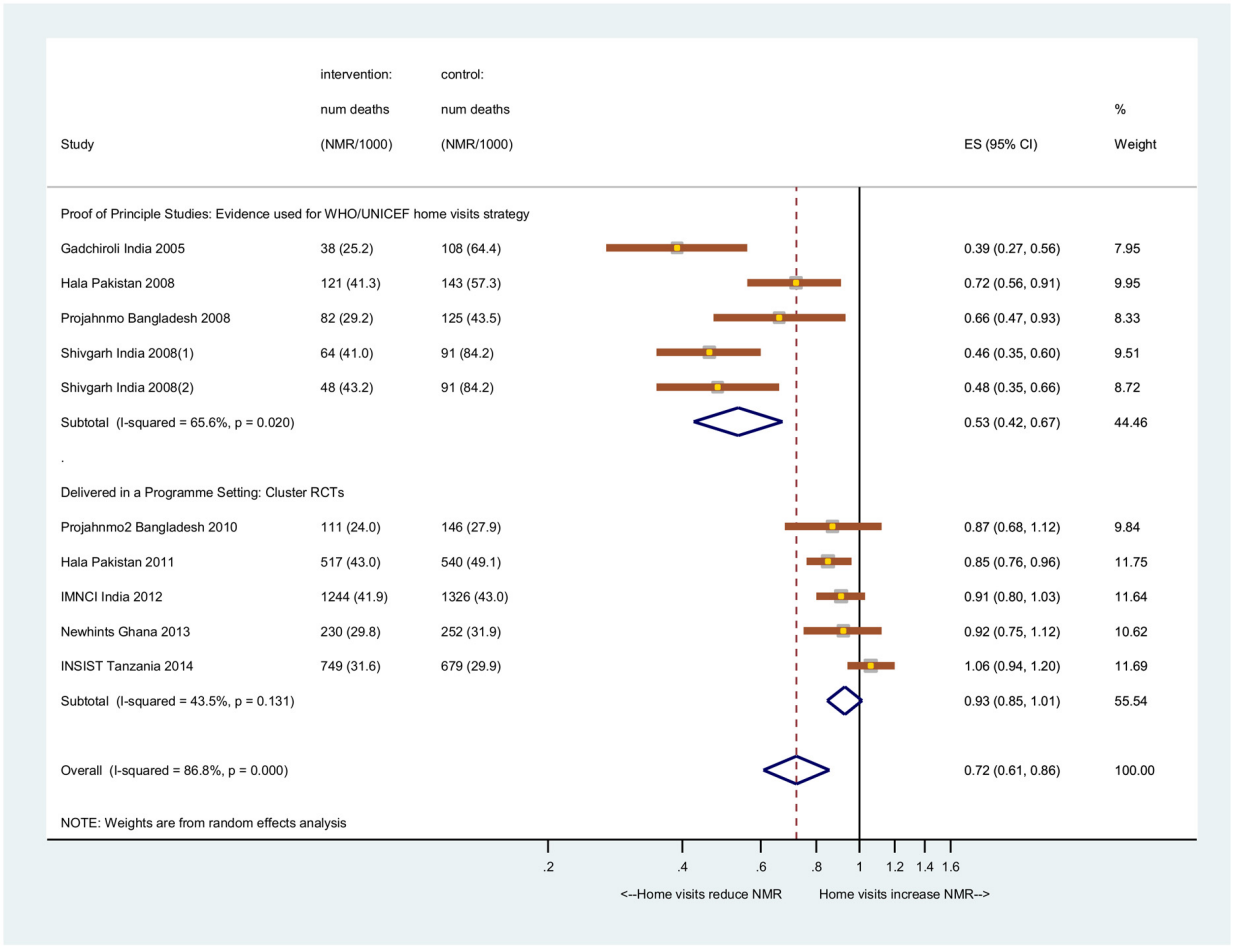
Meta-analysis of the effect of home visits on NMR. Data are the number of deaths (newborn mortality rate per 1,000 live births). Proof-of-principle studies: Gadchiroli, India, 2005 [4]; Hala, Pakistan [7]; Projahnmo, Bangladesh, 2008 [6]; Shivgarh, India, 2008 [5]; Projahnmo-2, Bangladesh [33]; Hala, Pakistan [31], Integrated Management of Neonatal and Childhood Illnesses, India [32]; Newhints, Ghana [9]; and Improving Newborn Survival in Southern Tanzania (INSIST). Shivgarh-1 = home visits only. Shivgarh-2 = home visits plus thermospot.

The updated meta-analysis showed no evidence that antepartum and postpartum home visits have an effect on neonatal survival in programme settings, based on three trials from Southeast Asia and two trials from sub-Saharan Africa: ours and Newhints, Ghana [9] (risk ratio of 7%; 95% CI -1%–15%) (test for heterogeneity *p* = 0.131, I^2 89%; 95% CI 78–92) (Fig 3).

## Discussion

This large cluster-randomised controlled trial in southern Tanzania assessing the effect of a home-based counselling strategy in pregnancy and postpartum found no evidence of an effect on neonatal survival. Neonatal mortality decreased from 35 to 31 deaths per 1,000 live births between 2004–2007 and 2010–2013. The intervention resulted in improved newborn care practices, particularly for exclusive breastfeeding in the first 3 d and delaying bathing, each of which improved by at least ten percentage points. Large secular increases in facility delivery from 41% in 2007 to 79% in 2013 were observed. Remarkably, almost 3 y after a 6-d volunteer training course given by district health teams, around 60% of pregnant women received a home visit by a volunteer supported through existing community structures [25]. However, early postpartum visits remained very low.

The lack of an impact on survival was unexpected, given the 12% reduction in neonatal mortality for a home visit strategy suggested by a recent systematic review of programmatic trials [9] and the 25% reduction proposed in the work of Lassi and Bhutta, which included studies evaluating community-based interventions [35]. Reasons could include low postpartum visit coverage. There was often a delay in volunteers receiving information about a delivery, and the distance to reach the woman’s home was another main barrier. Another reason for the lack of effect on mortality might be the relatively high coverage of recommended newborn care behaviours, including antenatal care and facility delivery. This context contrasts with that of Asian trials in which home-based counselling strategies had the most dramatic effects on neonatal mortality and both facility-based antepartum care and facility births were at a low level (S1 Table) [31–33].

In addition, our home-based approach did not include any clinical component such as identification and referral of sick newborns or antibiotic therapy. However, the increase in facility delivery alone should have had a large impact on mortality over time if common assumptions that this leads to improved care hold true [36]. Bhandari et al. reported an effect of home-based care on neonatal mortality in home births but not in women who delivered in a health facility [32]. Further, almost half of the deaths in our study were on the day of birth and likely due to intrapartum-related and preterm birth complications [37]. The potential for home visits to prevent such deaths is limited, but even after excluding these first-day deaths, we found no evidence of a survival impact. Our study did not include morbidity data [38] or information on the quality of intrapartum care, for which severe limitations are reported from elsewhere in Tanzania [39].

Our study has several limitations. First, the effects of the intervention may have reached comparison areas, giving an underestimate of the effectiveness of the intervention. Some volunteers may have visited women in comparison areas living close to ward boundaries or with family ties, because as the due date approaches some women stay with relatives. In addition, other groups supported home visit programmes with similar messages in small parts of the study area. The main focus of our facility-based quality improvement work was promotion of facility-based childbirth: this was implemented in 24 of 200 facilities (all of those in the Ruangwa district, with a further four in Mtwara Rural), with a balance between intervention and comparison wards. Second, recall of care practices and neonatal mortality could be prone to error. To mitigate the effects of this, information on care practices was only collected for live births in the year prior to the survey, and all analysis was based on an intention-to-treat approach. Thirdly, we did not collect information on stillbirths: misclassification of early neonatal deaths as stillbirths and vice versa is common. Fourthly, it was not feasible to mask participants. The intervention itself might have led to reporting bias, with women in the intervention groups reporting mortality or care practices differently than those in the comparison group.

The trial took place in a rapidly changing and dynamic environment where facility delivery almost doubled within 6 y from 41% at baseline in 2007 [18]. Although the intervention might have played some role in the increase, it is likely that other contextual factors were also important: just 1 y after implementation started, 65% of the comparison area births were in a health facility [26]. In part the increase could be due to improved communication and transport: less than 10% of households had a mobile phone in 2007 compared to 48% in 2013. The availability of motorcycle transport also increased substantially, and the focused national antenatal care programme promoting facility delivery might have contributed to the shift. Lastly, facility-based quality improvement programmes emphasising facility delivery, including our own in 24 facilities, have been ongoing in the study area, as well as focal home counselling work by different development agencies [40].

Our study illustrates some of the challenges of undertaking effectiveness evaluations of an intervention designed for scale in a rapidly changing context. Firstly, despite a robust, cluster-randomised design, we found strong evidence of behaviour change in the comparison group and cannot rule out that this is due in part to our intervention. Secondly, we found that although home visits in pregnancy were common, only 15% of women who delivered in a facility had a postnatal visit within 2 d of childbirth. Although disappointing, this result illustrates the importance of studying effectiveness: a large-scale programme in a similar context is unlikely to achieve high coverage of early postnatal visits. In addition, our effectiveness trial faced similar problems to other recent large-scale evaluations in terms of being overridden by health systems improvements [41], supporting the call by Victora and colleagues for more investments in national evaluation platforms [42].

Our results from the meta-analysis including five studies in programme settings found no evidence of a reduction of neonatal mortality (7% CI -1%–15%), which is at odds with the 45% impact (95% CI 37%–52%) for the proof-of-principle trials, all done in settings with limited access to facility-based health care services and high neonatal mortality (>45 deaths per 1,000 live births) [43]. Our updated estimate is also at odds with the estimated effect of 12% (95% CI 5%–18%) previously provided by Kirkwood and colleagues [9].

In a separate systematic review that examined intervention packages including home visits rather than home visits alone, the authors found insufficient evidence to draw any conclusion on the effect of postnatal home visits on neonatal mortality, reporting that any effect might depend on the context and the extent to which home visits can complement or replace facility-based newborn care [44]. Our results are in line with this conclusion. In a Cochrane review, Lassi and Bhutta discussed that the education, training, and support of community health workers differs between the studies, which might explain some of the differences observed in the effect of the interventions [35].

Despite moderate increases in newborn care behaviours associated with the intervention, neonatal mortality was similar in intervention and comparison areas, questioning the evidence base in support of home-based counselling [3,9]. Factors affecting intervention success include overall levels of recommended care practices, the extent to which women and families use facilities for preventive and curative care, NMRs, and the quality of care provided in facilities [45,46], suggesting a need for better knowledge on why and how interventions work and under which conditions they might achieve greater mortality declines before recommending them for wider implementation [47].

Our findings also give a stark reminder that demand and supply side strengthening should go hand in hand. The moderate decline in neonatal mortality contrasted with substantial improvements in newborn care practices, suggesting that improvement in the quality of facility care is of highest relevance in this setting. Our results thus support the recent shift to prioritize improvement in quality of facility-based care in Tanzania [48] and internationally [49].

## Supporting Information

S1 Data Internal monitoring data. (XLSX)

S1 Table Comparison of home-based counselling trials in programme setting in pregnancy and postpartum. (DOCX)

S1 Text Protocol of the Intervention. (PDF)

S2 Text Additional “per protocol” analysis. (DOCX)

S3 Text PRISMA checklist. (DOCX)

## Abbreviations

CI: confidence interval
INSIST: Improving Newborn Survival in Southern Tanzania
NMR: neonatal mortality rate
OR: odds ratio
PDA: personal digital assistant
RR: risk ratio
UNICEF: United Nations Children's Fund
