# Supplementary material for: Effectiveness of a Home-Based Counselling Strategy on Neonatal Care and Survival: A Cluster-Randomised Trial in Six Districts of Rural Southern Tanzania
Source: PLoS Med. 2015 Sep 29;12(9):e1001881. doi: 10.1371/journal.pmed.1001881 (PMC4587813; doi:10.1371/journal.pmed.1001881)
Supplement: S1 Table — (DOCX) [file pmed.1001881.s002.docx]

Web-annex table: Comparison of newborn care behaviours with other community-based trials in a programme setting (Newhints, Hala, IMCNI and Sylhet/Projahnmo2)

|  | Improving Newborn Survival in Southern Tanzania, INSIST | Newhints, Ghana  [[1](#_ENREF_1)] | Hala, Pakistan  [[2](#_ENREF_2)] | IMCNI, India,  [[3](#_ENREF_3)] | Sylhet/Projahnmo2, Bangladesh [[4](#_ENREF_4)] |
| --- | --- | --- | --- | --- | --- |
| Evaluation period | Mid-2010-mid 2013 | Nov 2008 – Dec 2009 | 2Feb 2006- March 2008 | Jan 2008-March 2010 | Feb 2005-April 2006 |
| Health care delivery characteristics | 1.2 million people, 200 health facilities including 6 hospitals. Mean distance from household to any facility 2.8 km and to any hospital 20 km in 2013 | 0.6 million people, 4 district hospitals, health centres in most of the sub-districts (number not given) and additional government health centres at community level | 0.6 million people, 22 basic rural health units, two rural health centres and 1 district hospital, established lady health worker (LHW) system of 1 LHW for 1400 population | 1.1 million people, 18 primary health centres | 0.5 million, 24 union health and family welfare centres, 1 private referral hospital in urban adjacent area which was excluded from study, mean travel time to hospital 1 hour |
| Implementation package | Behaviour-change counselling during 5 home visits (2 post-natal) by community volunteers with emphasis on breastfeeding, hygiene, and identification and extra care of low birthweight babies. For low birthweight babies 2 additional visits.  Identification of low birthweight babies through foot size.  Community and health facility sensitisation, quality improvement intervention in only 1 of 6 districts (24 facilities). | Behaviour-change counselling during 5 home visits through existing community based surveillance volunteers including assessment of sick babies, referral of sick or low birthweight newborns. Equipped with weighing scales for identification.  Traditional Birth Attendant (TBA), community and health facility sensitisation.  Hospital based newborn-care training | Counselling on antenatal care, iron folate during pregnancy, nutrition and rest, early breastfeeding, delayed bathing, cord care and recognition of sick newborns.  Collaboration with TBAs. Minimum of three visits, but encouragement for 4 additional postpartum visits  3-day training of TBAs (Dai) on basic newborn care including newborn resuscitation. | Promotion of newborn care practices through 3 postnatal visits + 3 additional visits for low birthweight babies.  Identification of sick newborns, referral or treatment if indicated.  Improved case management at community and health facilities based on Integrated Management of neonatal and childhood illnesses (IMNCI).  Health system strengthening through improved supervision and established community drug depots. Training /orientation of health staff on IMNCI. | Community health workers to make 5 home visits, trained on surveillance, essential newborn care, neonatal illness surveillance and management of illness based on a clinical algorithm adopted from the Integrated Management of Childhood Illness. TBAs received a 2-day orientation. |
| Community based mobilization | Sensitisation | Sensitisation | Community mobilisation through community volunteers, encouragement of community emergency funds, group education sessions, and video docudrama | Sensitisation with women’s groups | not mentioned |

| Coverage of essential newborn care practices | | | | | | | | | | | | | | | |
| --- | --- | --- | --- | --- | --- | --- | --- | --- | --- | --- | --- | --- | --- | --- | --- |
|  | **INSIST, Tanzania**  **2010-2013** | | | **Newhints, Ghana**  **2008-2009** | | | **Hala, Pakistan**  **2006-2008** | | | **IMCNI, India,**  **2007-2010** | | | **Projahnmo2, Bangladesh**  **2004-2006** | | |
|  | Inter-vention | Control | % -point Differ-ence | Inter-vention | Control | % -point Differ-ence | Inter-vention | Control | % -point Differ-ence | Inter-vention | Control | % -point Differ-ence | Inter-vention | Control | % -point Differ-ence |
| ANC 4 visits (Bangladesh at least 1) | 47 | 43 | 4 | 76 | 74 | 2 | 13 | 9 | 4 |  |  |  | 69 | 49 | 20 |
| Facility delivery | 82 | 75 | 7 | 68 | 68 | 0 | 54 | 44 | 10 | 28 | 34 | 6 | 20 | 17 | 3 |
| Saved money for delivery or emergency | 95 | 90 | 4 | 86 | 80 | 6 |  |  |  |  |  |  |  |  |  |
| Had plan in case of emergencies (home deliveries) | 70 | 63 | 7 |  |  |  |  |  |  |  |  |  |  |  |  |
| Clean hands for home delivery | 92 | 88 | 4 | 93 | 87 | 6 |  |  |  |  |  |  |  |  |  |
| Baby not bathed before 6 hours after birth | 91 | 80 | 11 | 41 | 29 | 12 | 50 | 27 | 23 | 85~ | 46~ | 38 | 78^ | 13^ | 53 |
| Breastfed within an hour of birth | 42 | 35 | 7 | 49 | 41 | 8 | 43* | 27* | 16 | 41 | 11 | 30 | 80 | 55 | 25 |
| Exclusive breastfeeding day 26-32 days | 79 | 69 | 10 | 86 | 80 | 6 |  |  |  | 78 | 37 | 41 |  |  |  |
| Practiced skin to skin care for prematurely born babies | 41.9 | 36.1 | 5.8 | 44 | 24 | 20 |  |  |  | 2# | 0 | 2 |  |  |  |
| Sick babies taken to hospital | 79.7 | 77.4 | 2.3 | 77 | 55 | 22 |  |  |  |  |  |  |  |  |  |
| Nothing put on the cord | 92 | 87 | 5 |  |  |  |  |  |  | 84 | 40 | 47 |  |  |  |
| Implementation strength | | | | | | | | | | | | | | | |
| At least one counselling visits in pregnancy | 59 | 4 | 55 | 72 |  |  | 44 | 26 |  |  |  |  | 93 |  |  |
| At least one counselling visits postpartum | 41 | 3 | 38 | 63 |  |  |  |  |  |  |  |  | 79 |  |  |
| Counselling within 24 hours |  |  |  | 20 |  |  |  |  |  |  |  |  | 69 |  |  |
| Counselling visit within 2 /3 days postpartum | 15 | 1 | 14 |  |  |  | 34 | 13 |  |  |  |  |  |  |  |
| Counselling visit within 10 days postpartum |  |  |  |  |  |  |  |  |  | 90 |  |  |  |  |  |

*****within 30 minutes, ~bathing delayed after 24 hours; ^bathing delayed after 3^rd^ day, # for all babies

1. Kirkwood B, Manu A, Tawiah-Agyemang C, ten Asbroek G, Gyan T, Weobong B, et al. (2013) NEWHINTS cluster randomised trial to evaluate the impact on neonatal mortality in rural Ghana of routine home visits to provide a package of essential newborn care interventions in the third trimester of pregnancy and the first week of life: trial protocol. Trials 11: 58.

2. Bhutta ZA, Soofi S, Cousens S, Mohammad S, Memon ZA, Ali I, et al. (2011) Improvement of perinatal and newborn care in rural Pakistan through community-based strategies: a cluster-randomised effectiveness trial. The Lancet 377: 403-412.

3. Bhandari N, Mazumder S, Taneja S, Sommerfelt H, Strand TA (2012) Effect of implementation of Integrated Management of Neonatal and Childhood Illness (IMNCI) programme on neonatal and infant mortality: cluster randomised controlled trial. BMJ 344.

4. Darmstadt G, Choi Y, Arifeen S, Bari S, Rahman S, Mannan I, et al. (2010) Evaluation of a cluster-randomized controlled trial of a package of community-based maternal and newborn interventions in Mirzapur, Bangladesh. PLoS One 5: e9696.
