# Supplementary material for: Effectiveness of a Home-Based Counselling Strategy on Neonatal Care and Survival: A Cluster-Randomised Trial in Six Districts of Rural Southern Tanzania
Source: PLoS Med. 2015 Sep 29;12(9):e1001881. doi: 10.1371/journal.pmed.1001881 (PMC4587813; doi:10.1371/journal.pmed.1001881)

STUDY PROTOCOL: Improving newborn survival in rural southern Tanzania: a cluster-randomised trial to evaluate the impact of a scaleable package of interventions at community level with health system strengthening

Authors (in alphabetical order): Jo Borghi[1], Simon Cousens[2], Yuna Hamisi[3], Claudia Hanson[3,4], Jennie Jaribu[3] , Fatuma Manzi[3] (co-PI), Tanya Marchant[4], Elibariki Mkumbo[3], Hassan Mshinda [5] (co-PI), Suzanne Penfold[4], David Schellenberg[4] (co-PI), Joanna Schellenberg[4*] (PI), Donat Shamba[3], Marcel Tanner [6,7] (co-PI)

[1] Department of Global Health and Development, London School of Hygiene and Tropical Medicine, London, UK

[2] Department of Infectious Disease Epidemiology, London School of Hygiene and Tropical Medicine,

[3] Ifakara Health Institute, Dar-es-Salaam, Tanzania

[4]Department of Disease Control, London School of Hygiene and Tropical Medicine, London, UK London, UK

[5] Tanzania Commission of Science and Technology (COSTECH), Dar-es-Salaam, Tanzania, Tanzania

[6] Swiss Tropical and Public Health Institute, Basel, Switzerland

[7] University of Basel, Basel, Switzerland

*corresponding author

Abstract

Child mortality has declined substantially in many countries including Tanzania, but newborn mortality remains high and around 3 million babies die every year in the first 28 days of life. Community-based approaches with home visits in the first week of life have shown great potential to reduce newborn mortality.

INSIST aimed[1] to develop, implement and evaluate an integrated, two-part strategy that combines interventions at community level with health system strengthening in rural Southern Tanzania to reduce newborn mortality. The community intervention focused around interpersonal communication through home visits in pregnancy and the early neonatal period by a village-based "agent of change". Key messages focused on hygiene during delivery, immediate and exclusive breastfeeding, and identification and extra care for babies born small because of low birth weight or prematurity. Extra care for babies born small included skin-to-skin care for small babies and referral to hospital for very small babies. The community intervention was implemented in six poor rural districts in Southern Tanzania, with 65 of the 132 wards within these districts randomized to receive the community intervention. In addition, a health system quality-improvement package was implemented in all health facilities of one district.

Data collection for the evaluation included i) a baseline household survey in 2007 of all 243,000 households in 5 of the 6 study districts to estimate baseline mortality and prevalence of newborn care behaviours, ii) an adequacy survey in 2011 in a representative sample of 5,000 households to estimate coverage of home visits and prevalence of newborn care behaviours, and iii) an endline household survey in 2013 in a representative sample of 200,000 households to estimate newborn and maternal mortality and prevalence of newborn care behaviours. The final analysis was based on "intention to treat", comparing newborn survival in babies born to women living in intervention compared to comparison wards.

The study is registered on clinicaltrials.gov, number NCT01022788.

Background

Every year 3 million babies around the world die during the neonatal period (the first 28 days of life). Although large improvements in child survival have been seen in the past ten years, newborn mortality has declined less than overall child mortality (under five years). In sub-Saharan Africa under-five mortality reduced by 47% (from 177 in 1990 to 98 deaths per 1000 live births in 2012) but newborn mortality only by 28% (from 45 in 1990 to 32 in 2012 deaths in 1000 live births) since 1990 [1]. Around 44% of all child deaths now occur in the neonatal period. Millennium Development Goal 4 – to reduce child mortality by two-thirds between 1990 and 2015 – will not be reached without further progress in reducing newborn mortality.

Effective interventions during the antenatal, peripartum and post-partum period could prevent most of these deaths, if implemented at scale [2]. However, newborns are often not reached through facility-based care during the most vulnerable period around birth, particularly in places where

[1] This protocol is written in the past tense throughout, although at the time of writing not all activities are complete

facility delivery is low. In addition postnatal care is poorly developed in the immediate postpartum period, and where available care is often of low quality.

At the time the INSIST study was planned, community-based approaches with home visits in the first week of life had shown great potential to improve newborn and maternal survival in Asia (see for example [3-5]). These home visits aimed at providing information on essential newborn care, newborn danger signs and the importance of referral for sick or premature newborns. The Lancet Neonatal Survival series published in 2005 suggested that 12-26% of neonatal deaths could be prevented by implementing high-coverage programmes of universal outreach and family-community care [6].

The reports from the trials in South Asia led to several randomised controlled trials in Africa evaluating community approaches to reduce newborn mortality. Two have been published recently. The intervention in the Newhints trial in Ghana comprised five home visits, two during pregnancy and three in first week of life, which led to an 8% (95% CI –12 to 25; p=0·405) decline in newborn mortality [7]. A meta analysis including this and previous trials from Africa and Asia suggested a 12% reduction in newborn mortality through home visits ante- and postpartum for sub-Saharan Africa and South Asia [7]. The MaiMwana trial in Malawi, using a factorial design of women's groups and health education by peer counsellors, also reported improvements in maternal and newborn health [8].

Tanzania is the largest country in East Africa with an estimated population of 48 million. Child mortality rates declined between 1990 and 2010 from 158 to 68 deaths per 1000 live births [9]. Less dramatic progress has been seen with newborn mortality where levels declined from 43 to 34 deaths per 1000 live births since 1990 [10].

The Tanzanian government is committed to reducing maternal, newborn and child mortality as outlined in the national road map [11]. This plan explicitly encourages community-based action and in particular mobilization of "community own resource persons" for newborn health, but the availability of these volunteers varies widely, as does their engagement for newborn health.

The country has a large network of primary health facilities and 68% of the population live within six km of a primary care facility [12]. The potential of this network is mitigated by the ongoing human resource crisis, particularly in rural areas. A study from Southern Tanzania [13] reported that compared to the national Tanzanian guidelines, health facilities in Lindi and Mtwara regions in 2004 had only 20% of the required prescribers and 14% of the required nurses employed. In addition absenteeism is high due to long and short-term training, sickness and leave. Only 41% of facilities had a prescriber (a medical officer, assistant medical officer, clinical officer or assistant clinical officer) available on the day of the survey.

The INSIST trial was developed against this background of a large network of primary facilities, a challenging human resource situation and extensive formative research on newborn care practices in the study area [14, 15]. The intervention was developed in close consultation with a national core group of advisors comprising members from the Ministry of Health & Social Welfare, the national coordinator for neonatal health, WHO, UNICEF and the Paediatric Association of Tanzania.

Objectives

INSIST aimed to develop, implement and evaluate an integrated, two-part strategy that combined interventions at community level with health system strengthening in rural Southern Tanzania.

The specific objectives were:

a) To develop and document a community-based package for improved newborn care, focussed around interpersonal communication through home visits in pregnancy and the early neonatal period by a village-based “agent of change” linked to existing village health volunteers.

b) To develop and document a quality improvement package for antenatal, intrapartum and postnatal care in health facilities.

c) To implement these strategies for improved newborn survival in such a way as to be both sustainable and scalable at national level.

d) To explore barriers, facilitators, and influencers involved in the community intervention from both health providers and community perspective and make recommendations for improvement.

e) To measure incremental costs and cost savings to the health sector and society associated with the interventions, and to predict the cost of integrating the programme into routine health service provision and of scaling-up.

f) To strengthen Tanzania’s capacity to develop and implement interventions to improve neonatal survival, and to undertake effectiveness evaluations.

g) To estimate the effect of the interventions on household behaviours related to newborn health.

h) To estimate the effect of the community-based package on newborn survival.

Methods

The INSIST study was implemented by a well-established collaborative group comprising Ifakara Health Institute, Tanzania, the London School of Hygiene and Tropical Medicine, United Kingdom and the Swiss Tropical Institute, Switzerland. Strong links were established with the district and regional health management teams in the four years prior to the start of the study. Five of the six study districts of INSIST were involved in a trial evaluating the effectiveness of intermittent preventive treatment of malaria in infants (IPTi)[16]. Health facility surveys and a large household census carried out as part of the IPTi study in 2007 were used in the formative research for the development of the INSIST interventions as well as providing baseline data for evaluation.

The study area

The study area was in Southern Tanzania and included all of Mtwara Rural, Newala and Tandahimba districts of Mtwara region and Lindi Rural, Ruangwa and Nachingwea districts in Lindi region. Approximately one million people lived in the area, mostly in small rural settlements and four smaller semi-urban district capitals.

The majority of the population in these districts lived by subsistence farming, or for those near the coastline, from fishing. Some income—although unreliable and volatile over time—was generated

from selling cashew nuts, particularly in Mtwara region (Tandahimba and Newala districts)[17]. Groundnuts and sesame were the other main cash crops. Most people lived in mud-walled or sun-dried brick houses. Iron roofs were increasingly available but thatched-roof houses remained common. Access to water was mostly from protected or unprotected public wells.

Both regions were among the least industrially developed in Tanzania. The main ethnic groups in Lindi and Mtwara regions were the Makonde, Mwera Makua and Yao, which share some cultural traits and are part of a wider belt of matrilineal people in Eastern and Central Africa. Most people spoke their local language, but Swahili was also widely spoken.

Maternal and newborn health and health care in the study area

The study area was served by a network of primary health facilities (196 dispensaries and 20 health centres), six hospitals and a further three hospitals just outside the district boundaries (status as of 2013). The first-line facilities (dispensary and health centres) offered a limited package of antenatal, intrapartum and postnatal care. However care for complications in pregnancy and childbirth was little-developed in first-line facilities [18, 19]. At hospitals, Caesarean section and blood transfusion services were available, but the availability of other emergency care interventions such as vacuum extraction or care for eclampsia was limited [20]. Emergency newborn care was introduced relatively recently in a limited number of facilities and as of 2011 most facilities lacked resuscitation equipment and trained providers to deliver essential newborn care [18].

Newborn and infant mortality were 43 and 76 per 1000 live births respectively in the years 2001-2004 [19]. In the years 2004-2007, newborn mortality reduced to 34 per 1000 live births (unpublished data from baseline survey). A total of 507 maternal deaths were reported between June 2004 to May 2007 in the baseline survey, giving an overall maternal mortality ratio of 712 per 100,000 live births (95% CI 652–777) [21].

In 2007, facility delivery was only 41% (9,046 out of 22,243 respondents). Most women delivered at home, approximately half with a relative or friend and the other half with a traditional birth attendant. Just over half (58%) of women who had delivered at home said they had made plans in case of an emergency during delivery. Also, just over half (58%) of women who delivered at home reported that the attendant wore gloves, only 46% of respondents reported that the birth attendant washed her hands before delivery and used soap [22].

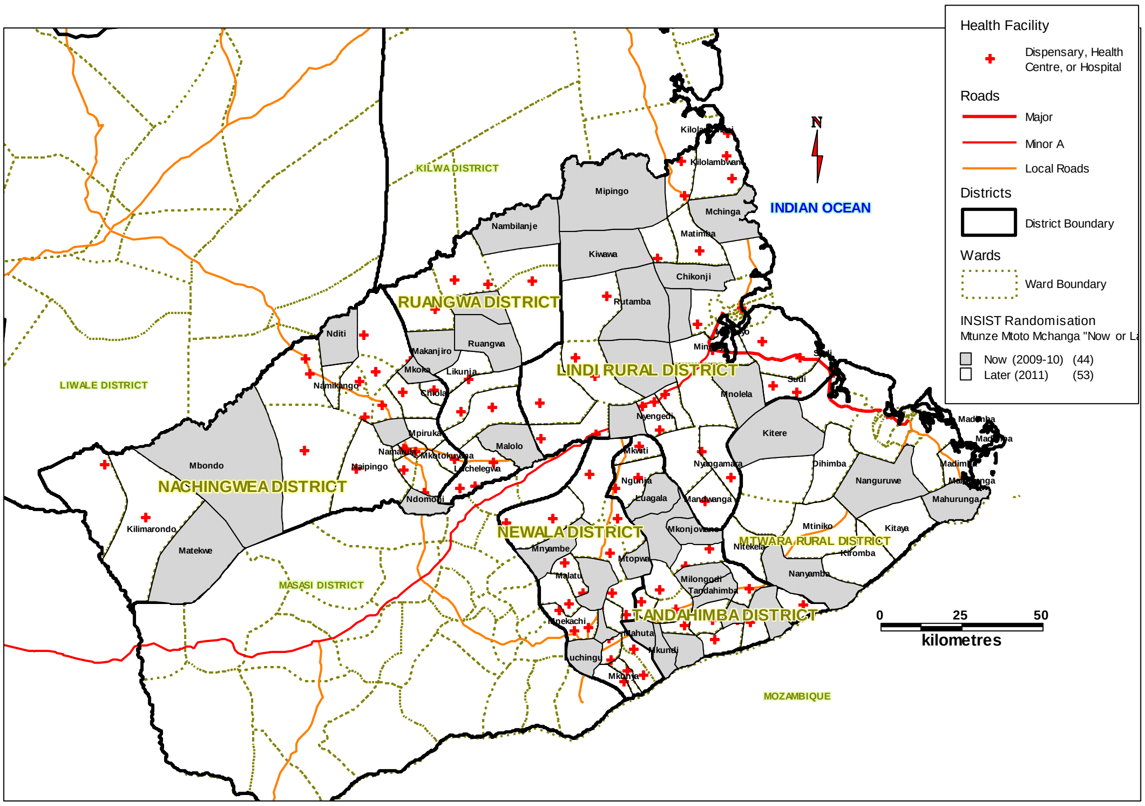

Figure 1: The study area showing the wards and health facilities

Overview of study design and methodology

The community-based newborn care package involves volunteers visiting women and their families at home, branded in Swahili as *"Mtunze Mtoto Mchanga"* (protect the newborn). This was evaluated using a cluster-randomised effectiveness design with changes in newborn survival as the main outcome. The unit of intervention was the ward, an administrative area below district level, with a median population of 6,933 people (Interquartile range 4,473-9,197) and approximately 2,500 households. Half of the wards were randomly selected in late 2009 to receive the intervention, the other half served as a comparison group.

Development and implementation of the community-based newborn care package

The intervention package was developed in partnership between the research team, Ministry of Health staff from local, regional and national levels, and other stakeholders. The implementation was led by government staff to ensure 'real-life' conditions and feasibility for scale-up. In addition, local health managers, the (district) Council Health Management Teams (CHMT) and the Regional Health Management Team (RHMT) were involved, particularly in training, and in development of the system for follow-up and support of the volunteers. The design of the intervention took the most recent Primary Health Care Policy into account [23].

Home visits by volunteers

The package focused on behaviour-change communication through a village-based "agent of change" called an *Mtunze Mtoto Mchanga* volunteer. These volunteers were recruited from their own

communities and were trained to make three home visits to women and their families during pregnancy and a further two visits, on the day of delivery and day 3 after delivery, and two additional follow up visits if the baby was low birthweight (Table 1). The volunteers also advocated within the village for assistance with emergency transport when needed, through community meetings and in meetings with village leadership.

Behaviour change communication focused on hygiene during delivery, including use of gloves, immediate and exclusive breastfeeding, and identification and extra care for babies born small or premature. For home births the volunteers were trained to measure foot size as a proxy for birth weight, and counselled the mother to adopt skin-to-skin care for small babies and for referral to hospital for very small babies[24]. Additional behaviours promoted included birth preparedness messages about the importance of delivery in a health facility and preparing clean cloths, soap, gloves, a clean blade, a clean cord tie, and money; delayed bathing of the baby; and putting nothing on the cord.

To support early home visits immediately after birth in the context of increasing facility deliveries, a delivery notification slip was introduced in early 2012. This notification slip was handed to the mother or family member by the facility staff on discharge or when a baby was brought to a facility soon after birth. The family was advised to take the notification slips to the volunteer so that she was alerted to the delivery. For home deliveries families were advised to inform the volunteer immediately after birth.

Materials used

The volunteers were provided with counselling cards for each family, a laminated counselling card for themselves, and a doll (known locally as a *bonto*) to support hygiene and breast feeding counselling. The counselling cards included pictures advising what to prepare for delivery, on hygiene, early and exclusive breastfeeding, about identification and extra care of low birthweight babies, and newborn danger signs (see card in annex).

In addition to the counselling material, the volunteers received “Mtunze”-T-shirts and umbrellas and locally-made cotton bags. They were also given identity cards. They had workbooks with a reminder of their training materials and their job descriptions and a register for each home visit.

The volunteers were also given referral slips, to be used when they referred a premature or low-birth weight baby to a hospital, as well as delivery notification slips, the latter from 2012.

Table 1: Timing of home visits, key messages & links to facility-based care

Visit	Timing	Key behaviours promoted	Additional behaviours promoted	Equipment
1	As soon as pregnant woman identified	• Birth attendant should wash hands and wear gloves	• Birth preparedness: preparing for facility delivery, saving money, preparing clean cloths, soap, clean blade for cutting & clean thread (or cord clamp) for tying cord, gloves for birth attendant	Counselling card
2	Four weeks after visit 1	• Early and exclusive breastfeeding	• Check on birth preparedness issues from previous visit	Counselling card
3	At the beginning of 9th month of gestation	• Early and exclusive breastfeeding including position<br>• In case of home birth:<br>o Birth attendant should wash hands and wear gloves, including while tying and cutting the cord<br>o Identification of low birth weight babies using foot size as a proxy<br>o Immediate referral for very small or premature babies, and those who don't cry<br>o Skin to skin care for small babies	• Check on birth preparedness issues from previous visits<br>• Warmth: immediate drying and wrapping, delayed bathing, not washing off vernix<br>• Danger signs for sick newborns<br>• In case of home birth, cord should be cut with clean blade and tied with clean thread	Counselling card with doll
4	Day of childbirth	• Observe breastfeeding and counsel on positioning<br>• Reminder of exclusive breastfeeding<br>• In case of home birth:<br>o Identification of low birth weight babies using foot size as a proxy<br>o Immediate referral to hospital for very small or premature babies<br>o Skin to skin care for small babies	• Check on warmth and knowledge of danger signs (as above)<br>• Put nothing on cord	Counselling card – measure foot size
5	Third day after delivery	• Observe breastfeeding and counsel on positioning<br>• Reminder of exclusive breastfeeding	• Put nothing on the cord<br>• Repeat: Danger signs for sick newborns<br>• Postnatal care 7d post-partum	Counselling card
1st extra visit for small baby	Day after visit 5	• Skin to skin until the baby doesn't want to be carried skin to skin		Counselling card
2nd Extra visit for small baby	Day after visit 6	• Skin to skin until the baby doesn't want to be carried skin to skin		Counselling card

Selection and training of volunteers

Volunteers were selected from villages within the intervention clusters. Each village includes approximately 350 households and it was expected that approximately 30—40 babies will be born each year.

The selection and training of volunteers was prepared through sensitization meetings of key health management staff (RHMT, CHMT) and ward level political and administrative leaders. In each of the villages in the intervention wards six female volunteers aged 20-35 years were selected. The selection process was initiated by an advertisement where interested women with primary school education, able to read and write, resident in the village and within the stipulated age range were requested to apply. The village leadership selected six candidates from these applicants. All these candidates were invited for a pre-test before training, after which the best four were trained for five days followed by a field day to connect the volunteers with traditional birth attendants and the administrative and political leaders. The training was followed by a competitive post-training test where two of the four trained volunteers from each village were selected to start working immediately after the training, whereas the other two served as 'reserve' volunteers. Often the selected members had been working as volunteers with other projects, such as having worked as Community Own Resource Persons (CORPS)[2] or Village Health Workers. However, experience of or engagement with other volunteer projects was neither a selection criterion nor encouraged during the selection process.

The training was initiated by a training of trainers (TOT) where members of the CHMT were trained by RHMT and project staff on birth preparation, danger signs, essential newborn care practices and counselling skills. After this, the four selected volunteers from each village were trained by their own CHMTs over a period of six days, approximately one ward at a time, over the first half of 2010.

Supervision and support of volunteers

Two models of supervision were used during the course of implementation. In the first model, which was used from March to December 2010, a single person supervised each volunteer. These supervisors were staff members in charge of the reproductive and child health section of the nearest health facility. The second model, adopted from January 2011, involved two different supervisors for each volunteer, and was community-linked. Village leaders were responsible for community volunteer supervision and support, and facility staff provided technical input. The village leaders, who were salaried government workers, met with the volunteers monthly and discussed implementation-related issues. In addition the volunteers visited their facility supervisors once per month for exchange of experience and advice on technical issues.

Quarterly review meetings were held for each ward, bringing together (1) all the volunteers (2) the village leaders and (3) the staff member in-charge of reproductive health services from the health facilities in the ward. The meeting was chaired by the ward leader and attended by two CHMT members, and included sharing of experience, refreshing knowledge, technical inputs, reviewing progress and discussing challenges, strategizing and agenda setting for next quarterly review meeting.

---

[2] The community IMCI approach is to recruit 10 resource people (CORPS) in each village for home visits. However this program has very limited coverage in most districts of Tanzania, including the study area.

Key monitoring tools used were monthly checklist-type supervision monitoring forms where each supervisor reported supervision meetings, with the date, attendees, and the key issues discussed.

In addition, during the quarterly review meeting the work of each volunteer was summarised from their individual work books, including the number of pregnant women visited at home, their place of birth and the number of home visits in the early newborn period.

Incentives

During the initial 6-day training each volunteer was paid TSh 20,000 (US $12) per day. Volunteers received an allowance of TSh 20,000 during each quarterly review meeting. Each volunteer was given an ID card, certificates, a T-shirt, a bag and an umbrella over the period from 2010 to 2013.

Development and implementation of the health system quality-improvement package

The health system quality-improvement package was piloted in four facilities of one district and then implemented fully in a second district. Resource limitations prevented further spread. The package used a problem-solving approach based on the 'breakthrough' series collaborative approach developed by the Institute for Health Care Improvement [25]. Groups of staff from several health facilities of the same level of service delivery formed a group called a 'collaborative'. Under the guidance of a local mentor these quality teams were encouraged to use a structured approach using a Plan-Do-Study-Act framework and to work on issues affecting maternal and newborn health care services, such as encouraging child birth in a health facility through counselling at antenatal clinics. Teams were guided to find change ideas to improve implementation using resources within their own capacity. The teams then tested these ideas to see whether they truly improved implementation using their own data to indicate improvement. Collaboratives met regularly to share the learning of successful and unsuccessful ideas. The approach was developed after reviewing several models for improving quality of care at different levels of the health system in Tanzania [26, 27] and in close cooperation with national and international stakeholders with expertise in quality improvement.

At the start of the study other national stakeholders were planning to invest in health system infrastructure and training in the two study regions, improving basic and comprehensive emergency obstetric care (EmOC) and training IMNCI (WHO/EU, MoH/ADB, SCF-UK).

Baseline household survey, randomization and follow-up household surveys

Data collection for the evaluation included i) a baseline household survey in 2007 of all 243,000 households in 5 of the 6 study districts[3] to estimate baseline mortality and prevalence of newborn care behaviours, ii) an adequacy survey in 2011 in a representative survey of 5,000 households to estimate coverage of home visits and prevalence of newborn care behaviours, and iii) an endline household survey in 2013 in a representative survey of 200,000 households to estimate newborn and maternal mortality, prevalence of newborn care behaviours, and coverage of home visits.

The baseline household survey was a census done in five of the six study districts. This survey documented newborn survival in each ward from July 2004 to June 2007. All women of reproductive age in the study area were visited and those agreeing to take part were asked about all births in the

[3] Baseline data was not collected in the 6th district because prior to 2009 the study team had no funding to work in the 6th district.

previous five years,[4] including dates of birth and dates of death for children who had died. In addition to the assessment of newborn survival, verbal autopsies were done on a subsample of newborn deaths, using the INDEPTH network VA questionnaire. This survey was funded by the Bill & Melinda Gates Foundation through the IPTi consortium (www.ipti-malaria.org). Following this survey the wards were stratified according to newborn survival, and restricted randomisation used to allocate each ward to community-based-intervention or comparison arms. The restricted randomisation assured balance between the two arms with respect to overall population size, district, and baseline newborn survival.

The adequacy survey in 2011 included a sample of around 5,000 households to evaluate newborn practices. Differences between intervention and comparison groups in coverage of essential newborn care and behaviours were evaluated.

The endline household survey in 2013 assessed newborn survival (the primary endpoint) in the 65 intervention and the 67 comparison wards. In addition we estimated i) the effect of the community intervention on pregnancy and newborn care behaviours, including antenatal care, place of childbirth, and immediate newborn care indicators, ii) the coverage of volunteer home visits in pregnancy and the early newborn period and the timing of the post-natal visits, in both intervention and comparison areas, iii) pregnancy-related mortality in the 4 years prior to the survey, and iv) balance between the two groups in contextual factors.

Sampling procedures

For the baseline household surveys, all households in 5 of the 6 districts in the study area were visited in 2007. For the follow-up adequacy household survey in 2011, a cluster sample of 5,000 households was drawn. A single cluster of 40 households was selected from each ward randomised. A modified EPI-type sampling procedure was used to ensure that every household in selected wards had an equal probability of selection[19] .

The 2013 endline household survey included around 200,000 households. The primary objective was to compare newborn survival in intervention and comparison wards. The primary sampling unit (PSU) was the sub-village (*kitongoji* in Swahili) with a median population of roughly 100 households according to information from village leaders. We selected all sub-villages in the smaller wards (estimated to have 1800 households or less) and a sample of 20 sub-villages in the larger wards, chosen with probability proportional to estimated size. We included all households in a sub-village if local leaders estimated a sub-village size less than 131. For larger sub-village we used segmentation to limit the sample size in each PSU to a maximum of 131 households. This approach resulted in an average of approximately 94 households per sub-village.

Sample size

For the estimated effect of the community-based intervention on newborn survival, the number of wards needed in each group, c, is given by the following formula [28]:

$$c = 1 + f\,[\,2p(1-p)/n + k^2\,(p_0^2 + p_1^2)\,]\,/\,(p_0 - p_1)^2$$

[4] To ensure completeness of data in the previous 2-3 years, we asked about births in the previous five years.

where p_1 and p_0 are the expected proportions in the intervention and control arms, respectively, and n is the number of births in each ward (assumed equal in all wards). The factor f depends on the power; k is the coefficient of variation in ward mortality rates, and has been estimated using the approach described by Hayes and Bennett [29].

The following assumptions were made:

Neonatal mortality rate = 34 per thousand live births
Size of effect to be detected: 15% reduction in neonatal mortality
Two-sided alpha = 5%
Coefficient of variation in ward mortality rates (SD/mean) = 0.21
Births per ward per year= 101
Years of follow-up=3
Number of wards in each group = 65

We estimated the study to have over 76% power to detect a 15% reduction in neonatal mortality rates. A range of power calculations was done for variations in the assumptions above, and is available from the authors on request.

Conduct of the surveys

The questionnaire

For all three surveys a modular questionnaire was administered. The household listing module included identifiers for the *kitongoji*, the name of the household head, a household number and geographical position. The household module included information on all members of the household, their dates of birth, education, occupation and ethnic group. Information was also collected information on household assets including ownership of a radio, a bicycle, a phone, animals and housing characteristics as proxy markers of household socioeconomic status. The 2007 and 2013 surveys included information about deaths in women aged 13-49 in the household in the past three years, and whether the woman was pregnant, giving birth, or had given birth in the two months before her death.

All women of reproductive age (13–49 years) were asked about live births in the five years prior to the survey, whether the child is still alive, and dates of all demographic events. The pregnancy, childbirth care, postpartum and newborn care module for all live births in the year before the survey included data on pregnancy care, counselling, use of ANC, malaria control, knowledge of danger signs, preparation for childbirth, place of childbirth, attendant, hygiene, thermal care, breastfeeding within one hour of birth, and other essential newborn care. In addition women were asked about the number and timing of home counselling visits, contact with care providers, care-seeking for illness, cord care, breastfeeding, and hygiene. The questionnaires were programmed into handheld computers (Personal Digital Assistants (PDAs)) including quality assurance mechanisms such as skip patterns, range and logical checks.

Sensitization and consent

A few weeks before each survey was undertaken, the aims and modalities of the survey were introduced during sensitization meetings to local administrative and political leaders. These sensitization meetings were also used in 2007 and 2013 to update the list of sub-villages and number

of households per sub-village. In all surveys the sensitizer/mapper visited the selected sub-villages and households a day before the main survey team to select and map the households according to a standard operating procedure, and leave a letter with every household head to invite them to participate. The following day a member of the team of interviewers read out the invitation letter, which included the consent form, and requested written informed consent from household heads according to a standard operating procedure.

Logistics

The conduct of all three surveys was structured around teams, each with seven interviewers, a supervisor, a sensitiser/mapper (who visited each sub-village one day before the rest of the team) and a driver. Each interviewer interviewed an average of 15 households per day. In 2007 and 2013 groups of 4-6 teams formed a platoon, led by a platoon commander.

Data collection and recording

All questionnaire data were collected using PDAs, and GPS coordinates were collected using hand-held GPS units or smart phones. Data safety was assured through backups to an SD-card at the end of each interview module, daily uploads from all PDAs to a supervisor's laptop, and daily backups from laptops to CD or SD cards. Data quality control processes included accompanied interviews, random repeat interviews with comparison to the original, visits by a supervisor to every household reported to be absent, and reconciliation of hand-written daily work summaries prepared by the interviewers with computer-generated daily work summaries after synchronizing the data on the supervisors' laptop and before leaving the village. Over the longer surveys, comparisons were made of data collected by different teams and interviewers concerning absenteeism, refusal and time spent on each module, with feedback to supervisors as appropriate. The study team used this approach in previous household surveys [30].

Analytical methods for endline survey

Newborn survival was estimated as the proportion of all live births that survive to 28 days, for children born in the three year period from 1 July 2010 to 30 June 2013. Baseline newborn survival, for those wards where baseline data were available, was estimated using the same approach, with a two-year reference period from 1 July 2005 to 30 June 2007.

For all wards, the two groups were compared with respect to educational level of mothers, ethnic group, parity, and household wealth status. For the 114 wards with baseline data available, the two groups were compared with respect to neonatal mortality rate in 2005-7 and facility births in 2006-7.

Primary analysis

The analytical approach was "intention to treat": children born to women interviewed in intervention wards were allocated to the intervention group, and those born to women interviewed living in comparison wards allocated to the comparison group. Analysis was based on random effects logistic regression to account for the weighting and the cluster-randomised design. Given the large number of clusters randomised this individually-based approach was more efficient than cluster-level summaries, making better use of all available information. Quadrature checks were carried out, and in the event that these failed, Generalised Estimating Equations (GEE) and robust standard errors

used instead. Analysis was carried out both with and without adjustment for potential confounders (mentioned above), with the estimated effect of the intervention presented as an odds ratio together with a 95% confidence interval.

Secondary analyses

Statistical tests to check for evidence that the effect varied by any of the potential confounding variables were carried out and results presented for those where the effect was unlikely to be due to chance. Potential confounders included ward-level averages of socio-economic status (wealth quintiles), ethnic group, baseline facility births and baseline newborn care behaviours. A p-value of 0.01 was applied for this analysis.

In addition, using routine monitoring data and survival impact survey data, a dose-response analysis was conducted to test the hypothesis that mortality changes were greater in wards with higher home visit coverage.

Evaluation of the health system quality-improvement intervention

To estimate the effect of the health system quality-improvement intervention, changes over time in the neonatal mortality rate in areas with, and without, this intervention were compared, adjusting for contextual factors that changed over time. A similar approach was used using facility births as an endpoint.

Health facility surveys

Two health facility surveys were carried out, in 2009 and 2011, to track process measures related to the quality-improvement intervention, and to assess the structure and function of the health system. Process measures included availability and attitudes of trained and untrained staff, availability of equipment and supplies for issues related to antenatal, intrapartum and newborn care, and review of health management information system (HMIS) records regarding antenatal, childbirth, and newborn care. An initial plan to include morbidity monitoring in selected health centres and hospitals was dropped due to resource constraints.

Costing

The cost-effectiveness study adopted the widely accepted framework and methods of economic evaluation [31, 32]. As far as possible, a societal perspective was adopted for the inclusion of costs and benefits of the interventions. The methods for costing the intervention were adapted from those of COIN, the costing tool developed by Saving Newborn Lives (SNL) and the South Africa Medical Research Council in order to support comparability across the SNL interventions and study sites. Data collection instruments were adapted to the local context and to the specific interventions developed in Southern Tanzania.

Costs included covered 1) the resources used to deliver the interventions within the health system and at community level; 2) costs incurred at the household level of receiving the interventions; 3) costs to families and the health system of treatment of sick newborn babies and neonatal deaths. Costs were presented in both economic and financial terms. Standard cost-effectiveness estimates (cost per death averted, cost per life-year gained) were carried out. We also reported costs in terms

of processes (eg. cost per home visit, cost per volunteer trained), and information about the cost structure, with a focus on the replicability and sustainability of the intervention rather than its cost-effectiveness per se.

Ethical issues

Adequate information about the project was made available to national, regional and district health authorities to enable them to decide whether or not they wish to be involved. The proposal and survey design were presented at meetings of democratically elected councillors from each of the proposed wards in the study area in order to explain the nature of the project to them. Only wards whose leaders consented to participation were randomised. Individual written informed consent was not sought from pregnant women living in wards randomised to receive the community-based newborn care intervention, although families were free to decline the home visits.

The study complied with the International Ethical Guidelines for Biomedical Research Involving Human Subjects [33]. For every household survey, an information sheet about the survey was drawn up in Swahili, explaining why it was being carried out, by whom, and what it would involve. This was used to introduce the survey to the community leaders. A standard explanation and introduction was read out to each household head before individual informed consent was sought. Respondents were then asked if they had any questions and whether they agreed to take part in the study. All efforts were made to maximise participant confidentiality. The surveys involved no risk to participants. Any participants with an acute illness were referred to a health facility.

Ethical clearance was obtained from the Medical Research Co-ordinating Committee of the National Institute of Medical Research through the Tanzanian Commission on Science and Technology. Ethical approval was also given by the Ethical Committees of Ifakara Health Institute and the London School of Hygiene and Tropical Medicine. A Data and Safety Monitoring Board (DSMB) was formed to review study procedures, analytical plans and monitor data collection. The study followed the principles of GCP.

Dissemination plan

The Ministry of Health and key national stakeholders were closely involved in the design and inception. Throughout the study regular feedback was given at district, regional and national level. At a local level results were shared with community leaders and health managers through district and regional-level meetings. At national level results were shared with the core group of stakeholders and then with a broader group of stakeholders through half-day meetings. Results were also disseminated as part of the annual Health Sector Review. Presentations were made at relevant national and international conferences. Results were also written up for publication in peer-reviewed journals.

Acknowledgements

The study was primarily funded by the Bill & Melinda Gates Foundation through Saving Newborn Lives. Co-funding was received from the Laerdal Foundation, from UNICEF Tanzania for the extension to Mtwara Rural district, and from the Batchworth Trust for work on foot size as a proxy for birthweight.

We thank the national core group of key stakeholders, the regional and district health authorities as well as village and wards authorities for their engagement and support.

References

1. UN Inter-ageny Group for Child Mortality Estimations, *Levels and Trends in Child Mortality. Estimates developed by the UN Inter-agency Group for Child Mortality Estimation*. 2013: UNICEF, WHO, The World Bank, and United Nations.
2. WHO, The Aga Khan University, and PMNCH, *Essential Interventions, commodities and guidelines. A global review of key interventions related to reproductive, maternal, newborn and child health (RMNCH)*. 2011: Geneva.
3. Manandhar, D.S., et al., *Effect of a participatory intervention with women's groups on birth outcomes in Nepal: cluster-randomised controlled trial.* The Lancet, 2004. **364**(9438): p. 970-979.
4. Baqui, A.H., et al., *Effect of community-based newborn-care intervention package implemented through two service-delivery strategies in Sylhet district, Bangladesh: a cluster-randomised controlled trial.* The Lancet, 2008. **371**(9628): p. 1936-1944.
5. Bang, A., et al., *Neonatal and Infant Mortality in the Ten Years (1993 to 2003) of the Gadchiroli Field Trial: Effect of Home-Based Neonatal Care.* J Perinatol, 2005. **25**(S1): p. S92-S107.
6. Darmstadt, G.L., et al., *Evidence-based, cost-effective interventions: how many newborn babies can we save?* The Lancet, 2005. **365**(9463): p. 977-988.
7. Kirkwood, B.R., et al., *Effect of the Newhints home-visits intervention on neonatal mortality rate and care practices in Ghana: a cluster randomised controlled trial.* The Lancet, 2013. **381**(9884): p. 2184-2192.
8. Lewycka, S., et al., *Effect of women's groups and volunteer peer counselling on rates of mortality, morbidity, and health behaviours in mothers and children in rural Malawi (MaiMwana): a factorial, cluster-randomised controlled trial.* The Lancet, 2013. **381**(9879): p. 1721-1735.
9. WHO and UNICEF. *Building a future for women and children. The 2012 report*. Countdown to 2015. Maternal, newborn and child survival. 2012; Available from: http://countdown2015mnch.org/documents/2012Report/2012-Complete.pdf.
10. Oestergaard, M.Z., et al., *Neonatal Mortality Levels for 193 Countries in 2009 with Trends since 1990: A Systematic Analysis of Progress, Projections, and Priorities.* PLoS Med, 2011. **8**(8): p. e1001080.
11. United Republic of Tanzania and Ministry of Health - Reproductive and Child Health Section, *The National Road Map Strategic Plan To Accelerate Reduction of Maternal, Newborn and Child Deaths in Tanzania 2008-2015. One Plan*. 2008: Dar-es-Salaam.
12. National Bureau of Statistics. United Republic of Tanzania, *Household Budget Survey 2007.* 2008: Dar-es-Salaam, Tanzania.
13. Manzi, F., et al., *Human resources for health care delivery in Tanzania: a multifaceted problem.* Human Resources for Health, 2012. **10**(1): p. 3.
14. Shamba, D., et al., *Clean Home-delivery in Rural Southern Tanzania: Barriers, Influencers, and Facilitators.* J Health Popul Nutr, 2013. **1**: p. 110-117.
15. Mrisho, M., et al., *Factors affecting home delivery in rural Tanzania.* Tropical Medicine & International Health, 2007. **12**(7): p. 862-872.
16. Schellenberg, J., et al., *Cluster-randomized study of intermittent preventive treatment for malaria in infants (IPTi) in southern Tanzania: evaluation of impact on survival.* Malaria Journal, 2011. **10**(1): p. 387.
17. Ministry of Finance and Economic Affairs, *The Economic Survey 2007, United Republic of Tanzania.* 2008: Dar es Salaam, Tanzania.

18. Penfold, S., et al., *Staff experiences of providing maternity services in rural southern Tanzania -- a focus on equipment, drug and supply issues.* BMC Health Services Research, 2013. **13**: p. 61.
19. Armstrong Schellenberg, J., et al., *Health and survival of young children in Southern Tanzania.* BMC Public Health, 2008. **8**: p. 194.
20. Hanson, C., et al., *Health system support for childbirth care in Southern Tanzania: Results from a health facility census.* Submitted to BMC Research Notes.
21. Hanson, C., et al., *Maternal mortality and access to obstetric care in rural southern Tanzania: results from a census of 226,000 households.* In manuscript.
22. Penfold, S., et al., *A Large Cross-Sectional Community-Based Study of Newborn Care Practices in Southern Tanzania.* PLoS ONE, 2010. **5**(12): p. e15593.
23. The United Republic of Tanzania. Ministry of Health and Social Welfare, *Primary Health Service Development Programm (MMAM) 2007-2017.* 2007: Dar-es-Salaam
24. Marchant, T., et al., *Measuring newborn foot length to identify small babies in need of extra care: a cross sectional hospital based study with community follow-up in Tanzania.* BMC Public Health, 2010. **10**(1): p. 624.
25. IHI, *The Breakthrough Series. IHI's Collaborative Model for Achieving Breakthrough Improvement*, in *Innovation Series 2003*, Institute for Healthcare Improvement, Editor. 2003: Cambridge.
26. Mbaruku, G. and S. Bergström, *Reducing maternal mortality in Kigoma, Tanzania.* Health Policy and Planning, 1995. **10**(1): p. 71-18.
27. UNFPA. *The Quality Improvement and Recognition Initiative (QIRI)*. Available from: http://www.unfpa.org/webdav/site/global/shared/documents/publications/2009/unfpa_povertycard_tan_en.pdf.
28. Smith and Morrow, *Field Trials of Health Interventions in Developing Countries, A Toolbox, 2nd edition*. 1996, London and Basingstoke: Macmillian Education.
29. Hayes, R. and S. Bennett, *Simple sample size calculation for cluster-randomized trials.* International Journal of Epidemiology, 1999. **28**(319-26).
30. Shirima, K., et al., *The use of personal digital assistants for data entry at the point of collection in a large household survey in southern Tanzania.* Emerging Themes in Epidemiology, 2007. **4**(1): p. 5.
31. Drummond, M., et al., *Methods for economic evaluation of health care programmes. Third edition*. 1997: Oxford University Press.
32. Gold, M., et al., *Cost-effectiveness in health and medicine*. 1996: Oxford University Press.
33. CIOMS/WHO, *International Ethical Guidelines for Biomedical Research Involving Human Subjects*. 1993: Geneva.

Ukiona dalili za hatari, tafuta matibabu haraka

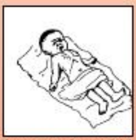
Kupumua kwa shida

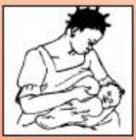
Kushindwa kunyonya vizuri

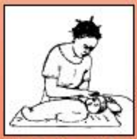
Joto jingi mwilini au baridi sana

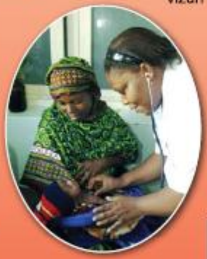

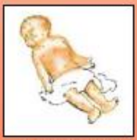
Ngozi kuwa na malengelenge yenye usaha

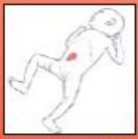
Kitovu chekundu kinatoa harufu au usaha

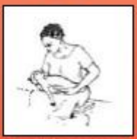
Mtoto kulegea sana au kulala kuliko kawaida au kuwa na degedege

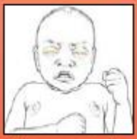
Macho yana usaha

WIZARA YA AFYA NA USTAWI WA JAMII

IFAKARA HEALTH INSTITUTE research | training | services

World Health Organization

Save the Children

unicef

Kadi ya Wazazi na Mtoto Mchanga

Jina la Mama			
Kijiji cha Mama		Kitongoji	
M/kiti wa Kitongoji			
Jina la Kliniki			
Jina la Muhudumu			
Mahali anapoishi			
Namna ya kumpata muhudumu			

Tarehe ya	1	2	3	4	5	Fuatilia 1	Fuatilia 2
Ahadi							
Kutembelewa							

Jiandae

- Kwa usalama zaidi jifungulie katika kituo cha huduma ya afya
- Tayarisha vifaa muhimu vitasaidia iwapo kuna dharura
- Weka akiba ya pesa kwa ajili ya usafiri wa dharura

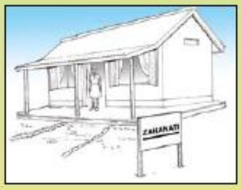

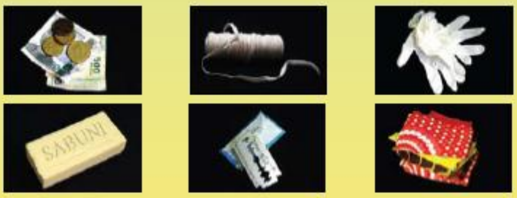

Usafi unapojifungua

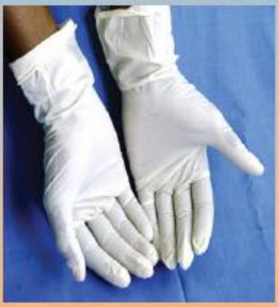

- Msaidizi wa uzazi atumie mipira kuzuia uambukizo
- Zingatia mazingira safi wakati wa kujifungua

Kwa mtoto mchanga mdogo kuliko kawaida

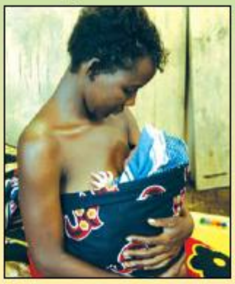

- Mtoto njiti apelekwe hospitali haraka
- Watoto wadogo kuliko kawaida watatambuliwa kwa kupimwa unyayo
- Mtoto mdogo kuliko kawaida abebwe ngozi kwa ngozi kwa muda mrefu

Matunzo ya mtoto mchanga

- Nyonyesha mtoto mara tu anapozaliwa
- Maziwa ya mama yanamtosha mtoto
- Kitovu cha mtoto kisiwekwe kitu chochote

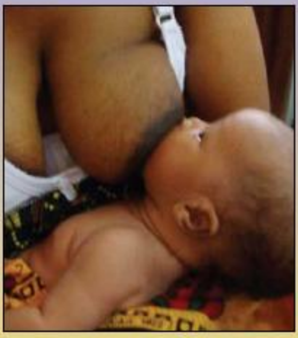

- Mtoto mwingine anazaliwa na utando mweupe kwenye ngozi. Hii ni kinga ya asili na humuongezea joto
- Mtoto aogeshwe kwa mara ya kwanza siku ya pili

Upimaji wa nyayo za mtoto mchanga

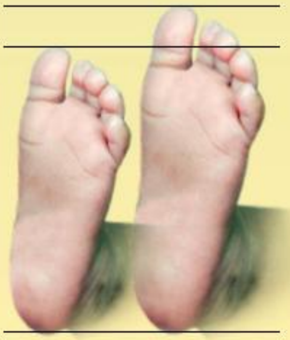

Supplement: S1 Text — (PDF) [file pmed.1001881.s003.pdf]
