# Supplementary material for: Effectiveness of a Home-Based Counselling Strategy on Neonatal Care and Survival: A Cluster-Randomised Trial in Six Districts of Rural Southern Tanzania
Source: PLoS Med. 2015 Sep 29;12(9):e1001881. doi: 10.1371/journal.pmed.1001881 (PMC4587813; doi:10.1371/journal.pmed.1001881)
Supplement: S2 Text — (DOCX) [file pmed.1001881.s004.docx]

**Rational for presenting the intention to treat and not per protocol analysis**

**Rationale for presenting the intention to treat and not per protocol analysis** 
We used for our estimation of the impact of the home-based counselling strategy an intention to treat analysis as recommended to avoid biased results. A ‘per protocol’ analysis, as often done in an individually-randomised trial, is an analysis restricted to participants who complied in full with the intervention. This is questionable for a cluster-randomised trial that is set-up to report the effect at population rather than at individual level. Moreover, results are likely to be biased as the reasons for non-compliance can be related to the intervention under study. We found evidence that mothers who reported having received and not received the intervention were statistically significantly different in terms of wealth status (p<0.001), district (p<0.001), and ethnic group (p= 0.020). In addition, since the death of a baby may affect recall of visits in pregnancy and the early newborn period, any ‘per protocol’ analysis on individuals is potentially subject to recall bias.

 Nevertheless, we did conduct a post-hoc analysis comparing survival in children born to mothers who reported receiving the intervention (at least 1 visit in pregnancy and one postpartum visit by a Mtunze volunteer, regardless whether the mother lived in intervention wards or not) with mothers who reported not receiving any visit (intervention and comparison wards). This analysis shows weak evidence of the effect of intervention on neonatal survival (OR 0.80; 95% CI 0.64-1.00; p=0.047; based on 127 and 280 neonatal deaths in mothers having received the intervention or not, respectively). The neonatal mortality rate was 31.5 (26.5-37.5) and 38.9 (34.6-43.8) for women who reportedly received and did not receive the intervention, respectively. The analysis adjusted for wealth status, district and ethnic group gave a similar result (adjusted OR of 0.8; 95% CI 0.6-1.0; p=0.026).
